# Three-Dimensional Heterocycles: New Uracil-Based Structures Obtained by Nucleophilic Substitution at the sp^2^ Carbon of Bromoisoxazoline

**DOI:** 10.3390/molecules19068661

**Published:** 2014-06-24

**Authors:** Misal Giuseppe Memeo, Francesco Lapolla, Bruna Bovio, Paolo Quadrelli

**Affiliations:** Dipartimento di Chimica, Univesità degli Studi di Pavia, Viale Taramelli 12, 27100 Pavia, Italy

**Keywords:** nitrosocarbonyl intermediates, 1,3-dipolar cycloadditions, bromonitrile oxide, nucleophilic substitution at sp^2^ carbon, uracils, thymine, X-ray analysis, nucleosides, β-turn inducers

## Abstract

The regioisomeric cycloadducts of bromonitrile oxide and *N*-benzoyl-2,3-oxaza-norborn-5-ene were easily prepared and elaborated into a novel class of uracil-based scaffolds. The key-synthetic step is the nucleophilic substitution at the sp^2^ carbon atom of the bromoisoxazoline three-dimensional heterocycles. The protocol to perform the nucleophilic substitution of uracil anions was optimized and adapted to the steric requirements of the substrates. A library of pyrimidine derivatives was prepared in very good yields and the products were fully characterized. They are proposed as nucleoside analogues and as synthons for β-turn motifs within PNA structures.

## 1. Introduction

**Scheme 1:**
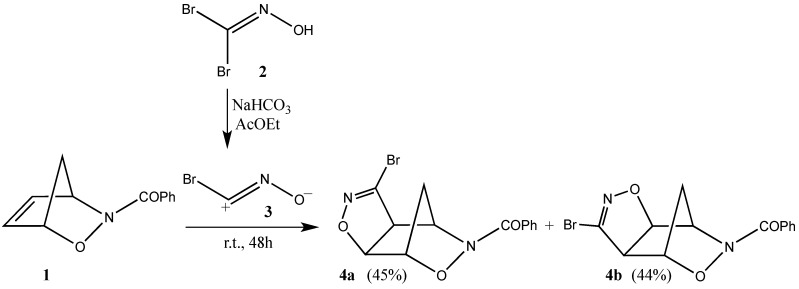
Cycloaddition reaction of bromonitrile oxide (**3**) to the *N*-benzoyl-2,3-oxazanorborn-5-ene (**1**).

A novel approach to useful precursors for the synthesis of isoxazoline-carbocyclic nucleosides was recently detailed starting from the readily available *N*-benzoyl-2,3-oxazanorborn-5-ene (**1**) and introducing a versatile functional group via a condensed bromoisoxazoline ring. These structures can be prepared through the efficient and *exo*-selective 1,3-dipolar cycloaddition reaction of bromonitrile oxide **3** (*in-situ* generated from the corresponding bromoxime **2**) to the 2,3-oxazanorbornene (**1**) affording the regioisomeric cycloadducts **4a**,**b** in good yields (Scheme 1) [1].

In previous studies the regioisomeric cycloadducts **4** were used as starting materials for the synthesis of aminols containing the β-hydroxynitrile functionalities derived from the isoxazoline ring opening [2]. They served as intermediates in the linear construction of uracil-substituted nucleosidic structures [3,4].

Nitrosocarbonyl (R-CONO) chemistry represents the key strategy of the reported syntheses because of the high dienophilic power of these fleeting intermediates and the synthetic potential of their hetero- Diels-Alder (HDA) cycloadducts of type **1**. Traditionally, nitrosocarbonyls are generated through periodate oxidation of hydroxamic acids and instantly trapped by dienes as reported by Kirby [5]. Recently, we developed two alternative entries to nitrosocarbonyls through the mild oxidation of nitrile oxides with *N*-methylmorpholine-*N*-oxide (NMO) [6,7,8,9,10].

**Scheme 2:**
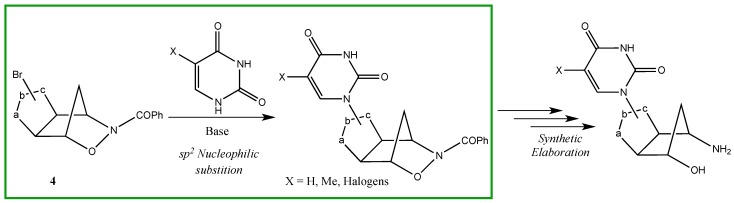
Synthetic strategy towards uracil derivatives.

In the planned efforts to expand upon the versatility of **1**[1,2,3,4,5,6,7,8,9,10], we were interested in varying the synthetic applications of the regioisomeric three-dimensional cycloadducts of the bromonitrile oxide to the oxazanorbornene **1**. The presence of the bromine atom on the nitrile oxide moiety suggests the exploration of the nucleophilic substitution (S_N_) reaction on a sp^2^ carbon atom [11]. We wish to report here a study regarding the reactivity of the regioisomeric cycloadducts **4a**,**b** in the presence of the *in-situ* generated anions of uracil bases (Scheme 2). The methodologies applied are discussed in the light of the reaction mechanism

The study also aimed to ascertain the scope and eventual limitations of this type of chemistry upon variation of the experimental conditions and the feasibility of the planned synthetic elaborations towards the preparation of novel β-turn inducers containing nucleobases for their insertion in Peptide Nucleic Acids (PNA) as well as the synthesis of new nucleoside analogues [12,13].

**Scheme 3:**
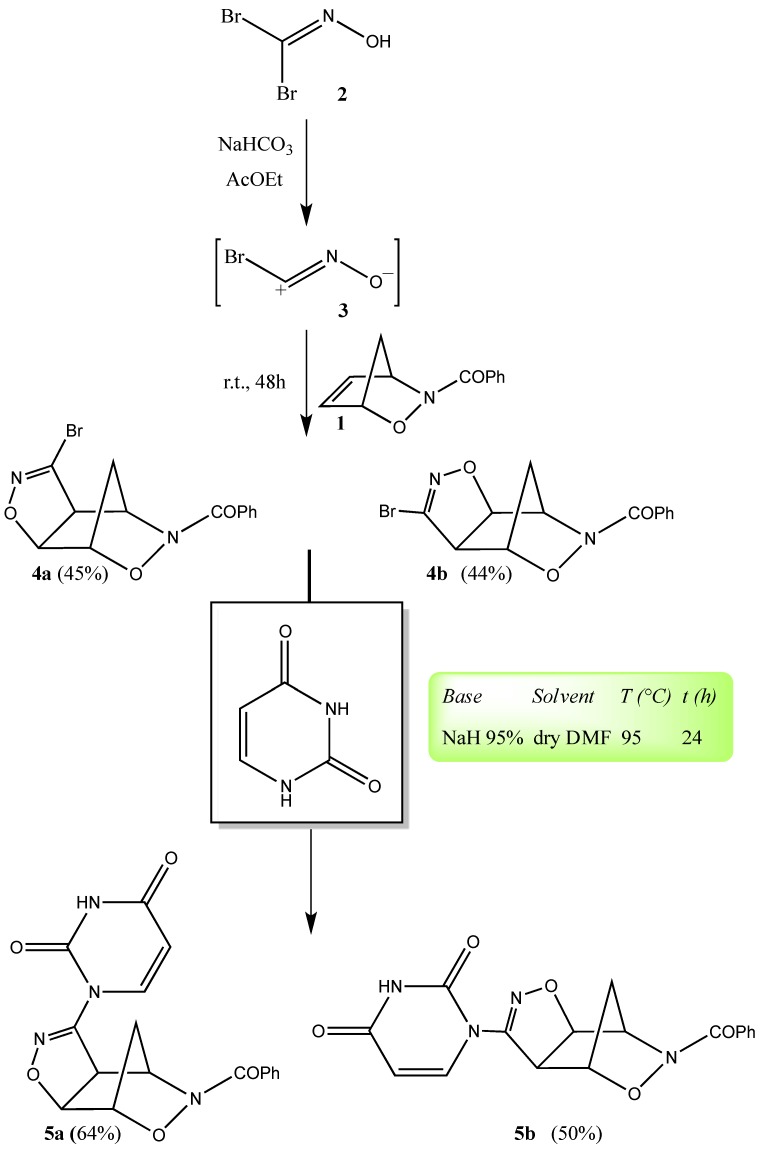
Synthesis of uracil compounds (**5a**,**b**).

## 2. Results and Discussion

The bromonitrile oxide **3** is generated *in situ* in an AcOEt/NaHCO_3_ solution from the corresponding bromoxime **2** [14], which is easily prepared by treating glyoxylic acid with hydroxylamine hydrochloride in the presence of bromine at 5 °C in dichloromethane [15]. Using *N*-benzoyl-2,3-oxazanorborn-5-ene (**1**) as dipolarophile, the 1,3-dipolar cycloaddition reaction smoothly occurs affording the regioisomeric compounds **4a**,**b** in 45% and 44% yields, respectively, which are easily isolated upon chromatographic separation (Scheme 3).

The structures of cycloadducts **4a**,**b** were assigned based on their analytical and spectroscopic data as well as upon comparison with previously prepared authentic samples [1]. The known exceptionally high reactivity and exclusive *exo*-selectivity of the cycloaddition reaction of **1** with the bromonitrile oxide **3** is akin to that of the related parent carbocyclic norbornene, which adds all types of reactants only on the *exo* face [16]. The origin of the norbornene selectivity is usually attributed to the relief of strain [17], geometric deformation of the double bond (pyramidalization due to torsional [18] and hyperconjugative effects [19]) as well as favourable staggering effects in the *exo* attack [20]. This fact pushed us to explore the insertion of a heterobase of choice *anti*-related to the oxaza-moiety of the cycloadducts **4a**,**b** through an sp^2^ S_N_ reaction as pivotal synthetic step of our strategy towards nucleoside analogues and β-turn motifs.

**Scheme 4:**
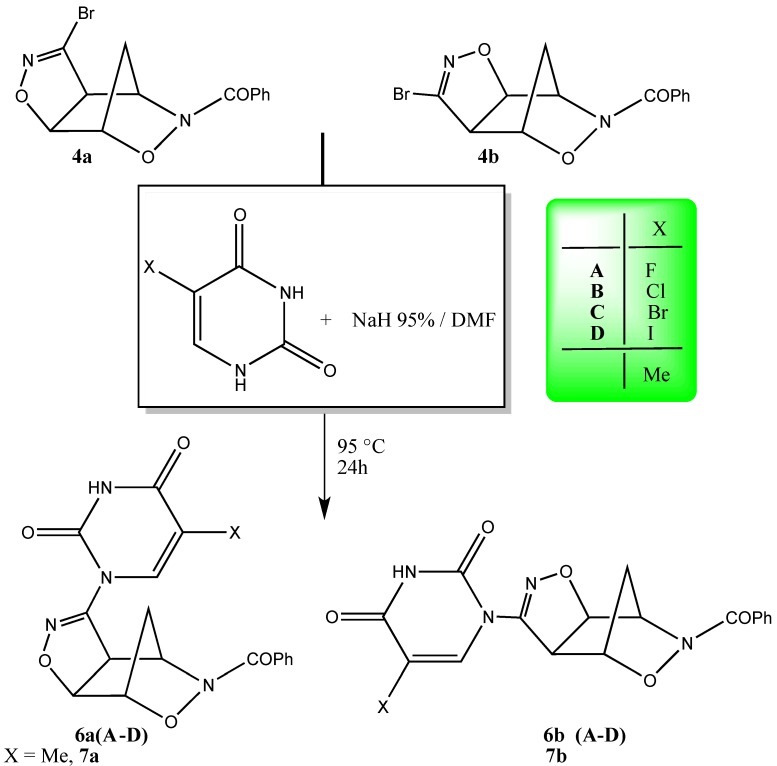
Synthesis of uracil derivatives (**6a**,**b**) and thymine compounds (**7a**,**b**).

The sp^2^ S_N_ reaction conditions and the synthetic method were first optimized using as benchmark reaction that between the two regioisomeric cycloadducts **4a**,**b** and to uracil as heterobase (Scheme 4). In the search for the best reaction conditions, we took advantage of previously reported results on an sp^2^ S_N_ reaction conducted on a bromoisoxazoline [21]. Upon addition of the regioisomeric cycloadducts **4a**,**b** to an anhydrous DMF suspension of the uracil anion, generated by treatment with NaH, under stirring at 95 °C, poor results were obtained in terms of final products **5a**,**b** (10%–12% yields, respectively) after 3 days of reaction. Moreover, great difficulties were encountered in the work-up procedures (emulsions) for the isolation of the products.

The reference authors themselves report the various efforts done to find the proper experimental conditions to perform the sp^2^ S_N_ reaction. However they did not detail the experimental procedure but indicated DMF as the best solvent to run the reactions along with the temperature and reaction time employed [21]. The choice of different solvents such as anhydrous THF or MeCN was soon abandoned since just the starting materials were recovered after the work-up and no reaction occurred. Then we tried to change base, temperature and reaction time. The sp^2^ S_N_ reaction failed when NaOH was used as well as running the reactions both at room temperature or upon heating, even after several days under stirring. Decomposition of the starting materials was also observed under microwave heating (MW) (DMF/NaH at 160 °C for 30 min).

We attributed the low yields of the adducts **5a**,**b** to the low concentration of the nucleophile. In order to have a high conversion of the uracil base into the corresponding anion, we used NaH 95% instead of the standard 70%–80% and performed the nucleophile *in-situ* generation under vacuum in a Schlenk tube. In an anhydrous Schlenk tube an excess of NaH 95% (2.1 equivalents) is suspended in anhydrous DMF (20 mg/mL) and vacuum is applied to get rid of the air. The uracil base is added portionwise and the suspension is left under stirring at room temperature for 30 min.

Finally, 0.8 equivalents of the regioisomeric cycloadducts **4a**,**b** were added and the mixture was heated at 95 °C under stirring for 24 h. The reaction was quenched by adjusting the pH to 7 by means of addition of aqueous 3:1 NH_4_Cl/NaCl and the water phase was extracted with dichloromethane (DCM). The adducts **5a**,**b** were purified by column chromatography under medium pressure (MPLC) and the yields were 64% and 50% for **5a** and **5b**, respectively (yields determined by HPLC-RP C-18 analysis on crude extracted solutions). The isolated yields were 44% and 30% for **5a** and **5b** of 98.5% purity after recrystallization, respectively.

**Figure 1:**
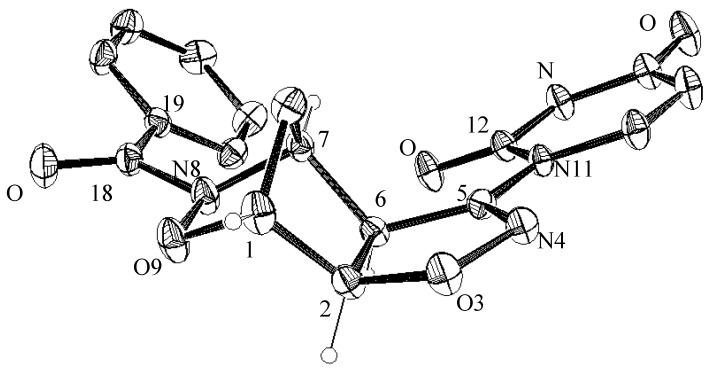
ORTEP plot of compound **5a** with atom labeling (ellipsoid at 20% probability).

The assigned structures of adducts **5a**,**b** rely upon the corresponding analytical and spectroscopic data. In particular, the vinyl protons of the uracil moiety in **5a** are found at δ 5.79 (d, *J* = 8 Hz, =CH-C=O) and at δ 7.83 (d, *J* = 8 Hz, N-CH=) while the singlet corresponding to the NH group is found at δ 11.70. Similarly, in compound **5b**, the vinyl protons of the uracil moiety are found at δ 5.79 (d, *J* = 8 Hz, =CH-C=O) and at δ 7.85 (d, *J* = 8 Hz, N-CH=) while the NH group singlet is found at δ 11.72. All the other signals of the three-dimensional isoxazoline-norbornane moieties are in the expected range and clearly support the reported structures (Scheme 3). A definitive confirmation of the structural assignments given above came from the X-ray structure of compound **5a** whose ORTEP view is shown in Figure 1. In the oxazanorbornane ring of the compound **5a** the sum of the angles at N8 345.0(2)° is consistent with the sp^3^ hybridization; the 2*pz* lone pair takes no part in π–bonding with the C=O group, indeed the bond distance N8-C18, 1.376(3) Å is longer than the shortened partial double bond (1.352(5) Å). The height of the pyramid with the nitrogen atom at the apex and the three atoms connected to it at the base is 0.230(3) Å. The atoms N8 C18 O18 C19 are almost coplanar: the maximum deviation from least–squares plane is 0.028(2) Å; the angle between the carbonyl group and the moiety C1 O9 N8 C7 is 36.1(2)°. Bond lengths in oxazanorbornane ring C-C (range 1.523(4)–1.534.1(3) Å) and N-C 1.477(3) Å are comparable with the values of the literature [22]. The six-membered ring of the oxazanorbornane is puckered with deviations from the least-squares plane in the range −0.569(3) + 0.305(2) Å. These ring exhibit a *boat* conformation with the parameters [23] *Q* = 0.985(2), φ = 60.4(1)°, θ = 90.3(1)°, (ideal conformation: φ = 60.0°, θ = 90°, respectively).

In the isoxazoline rings of compound **5a** the angle at N4 109.1(2)° is consistent with the sp^2^ hybridization of N4, whereby the 2*pz* lone pair takes part in the π–bonding within a part of the heterocyclic ring; indeed the bond distance N4-C5 1.270(3) Å shows a double–bond character. The internal angles at the nitrogen atoms O3-N4-C5 109.1(2)° in **5a** differ by 3.9°, justified by the different type of nitrogen. The deviation of atoms from the least–squares plane of isoxazoline rings are in the ranges −0.013(2) + 0.012(2) Å with a *T* conformations; the puckering parameters are *Q* = 0.020(3), φ = 132.2(2)° in **5a** (ideal value φ = 126°).

The pyrimidine ring in compound **5a** attached to the isoxazoline is nearly planar with a maximum deviation of −0.016(3) Å. This ring is tilted (with respect to the isoxazoline ring) by 5.3(1)° implying only a small conjugation. The orientation of the pyrimidine ring, due to the steric crowding the attached atoms is confirmed by the torsion angles N4-C5-N11-C12 −7.3(3)°, 12.2(4)°.

In order to ascertain the eventual limitations of this chemistry we extended the functionalization to the entire family of the halogenated uracils and thymine. Scheme 4 reports the reactions and the structures of the halogenated-uracil adducts **6a**,**b** (**A**–**D**) and the thymine adducts **7a**,**b** obtained by applying the above reported sp^2^ S_N_ reaction protocol. The positive results indicate that the methodology set-up for the uracil base can be easily transported to the other pyrimidine-type heterobases without any variation or adjustment when other derivatives are employed. In fact, as we can see from the data of Table 1, the overall yields are good, with small variations when moving from one substituent to another.

**Table 1:** Yields, physical data, relevant IR bands and diagnostic ^1^H-NMR signals of compounds **6a**, **b** (**A**–**D**) and **7a**,**b**.

Entry	Adduct 6a	Yield (%)	m.p. (°C) ^#^	IR (cm^−1^) ν_NH_	ν_C=O_	^1^H-NMR N-CH=CX	(δ, DMSO- *d*_6_) *J* (Hz)
1	**A**	46	193–198	3394	1733	8.24 (d)	7
2	**B**	53	180–189	3360	1734	8.23 (s)	
3	**C**	56	96–100	3200	1734	8.27 (s)	
4	**D**	57	110–115	3394	1632	8.23 (s)	
	**6b**						
5	**A**	64	95–100	3157	1723	8.24 (d)	7
6	**B**	76	98–105	3178	1732	8.24 (s)	
7	**C**	49	104–106	3121	1732	8.29 (s)	
8	**D**	57	162–164	3160	1720	8.24 (s)	
9	**7a**	45	215–217	3176	1715	7.69 (d)	1.81 (Me)
10	**7b**	55	220–222	3149	1710	7.69 (d)	1.82 (Me)

^#^ From diisopropyl ether/ethanol.

The structures of the adducts **6a**,**b** (**A**–**D**) and **7a**,**b** rely upon the corresponding analytical and spectroscopic data. Table 1 also reports the physical data (mps) and the frequencies of the IR bands corresponding to the uracil moieties inserted upon sp^2^ S_N_ reactions as well as the diagnostic ^1^H-NMR signals confirming the insertion of the uracil rings onto the tricyclic structures.

**Figure 2:**
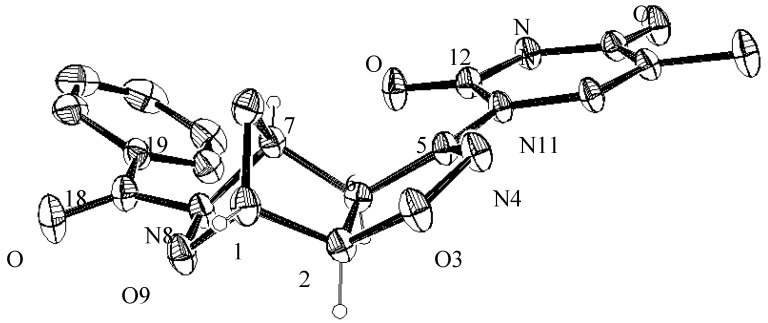
ORTEP plot of compound **7a** with atom labeling (ellipsoid at 20% probability).

Belonging the heterobases to the same family of the pyrimidine heterocycles, the changes in the NMR signals relative to the isoxazoline-norbornane moiety are negligible while the presence of the attached uracil units is clearly indicated by the vinylic protons as well as the NH groups found in the δ 12.05–12.25 range. A definitive confirmation of the structural assignments given above came from the X-ray structure of compound **7a** whose ORTEP view is shown in Figure 2.

In the oxazanorbornane ring of the compound **7a** the sum of the angles at N8 339.4(3)° is consistent with the sp^3^ hybridization; the 2*pz* lone pair takes no part in π–bonding with the C=O group, indeed the bond distance N8-C18 1.377(5) Å is longer than the shortened partial double bond (1.352(5) Å). The height of the pyramid with the nitrogen atom at the apex and the three atoms connected to it at the base is 0.249(3) Å. The atoms N8 C18 O18 C19 are almost coplanar: the maximum deviation from least–squares plane is 0.039(4) Å; the angle between the carbonyl group and the moiety C1 O9 N8 C7 is 40.9(2)° in compounds **7a**. Bond lengths in oxazanorbornane rings C-C (range 1.507(5)–1.540(5) Å) and N—C 1.487(4) Å in compound **7a**, are comparable with the values of the literature [22]. The six–membered ring of the oxazanorbornane is puckered with deviations from the least–squares plane in the range −0.306(5) +0.575(5) Å. This ring exhibits a *boat* conformation with the parameters [23] *Q* = 0.982(3)°, φ = 60.8(3)° and θ = 90.0(3)° (ideal conformation: φ = 60.0°, θ = 90°, respectively).

In the isoxazoline rings of the compounds **7a** the angles at N4 109.9(3)° is consistent with the sp^2^ hybridization of N4, whereby the 2*pz* lone pair takes part in the π–bonding within a part of the heterocyclic ring; indeed the bond distances N4-C5 1.272(4) Å shows a double–bond character. The internal angles at the nitrogen atoms O3-N4-C5 109.9(3)° and C7-N8-O9 105.3(3)° in **7a** differ by 4.6°, justified by the different type of nitrogen. The deviation of atoms from the least–squares plane of isoxazoline ring is in the ranges −0.032(3) + 0.031(3) Å, with a *T* conformations; the puckering parameters are *Q =* 0.055(4), φ = 126.9(3)° (ideal value φ = 126°).

The pyrimidine ring in compound **7a** attached to the isoxazoline is nearly planar with a maximum deviations of −0.061(4) Å; this rings is tilted (with respect to the isoxazoline rings) by 22.1(2)° respectively implying only a small conjugation. The orientation of the pyrimidine ring, due to the steric crowding of the attached atoms is confirmed by the torsion angles N4-C5-N11-C16 175.8(2)°, −165.0(4)°.

Finally, we have also scaled-up the protocol (from 250 mg to 2.5 g) performing the reactions with a larger amount of starting materials in order to have the required quantities of all the reported compounds to carry on with the synthetic elaborations towards the final targets. Yields did not change significantly (±2%) remaining in the reported range and the work-up procedures worked nicely, allowing for the easy isolation of the desired compounds.

The synthetic pathway shown in the Scheme 3 and Scheme 4 represent the pivotal step as well as the most delicate and somewhat difficult in the approach to our final goals. In fact, the first attempts to perform the sp^2^ S_N_ reaction failed and the method had to be properly set-up to be adapted to our tricyclic substrates.

The S_N_ reaction on a sp^2^ carbon atom is a transformation of relevance to organic synthesis, industrial applications as well as biochemistry [24]. Recent computational studies shed some light on the sp^2^ S_N_ reaction in comparison with the mechanism followed by the nucleophilic aromatic substitution, S_N_Ar, and the nucleophilic aliphatic substitution, S_N_2 [25].

Contrary to the aliphatic version of the S_N_ reaction, the sp^2^ S_N_ reaction on vinyl (V) carbons can follow different pathways. A common one is the Addition-Elimination route (A-ER); if electron-withdrawing groups are located on the α-carbon atom, a stable anion represents the intermediate of the reaction and the successive elimination affords the substitution product.

**Scheme 5:**
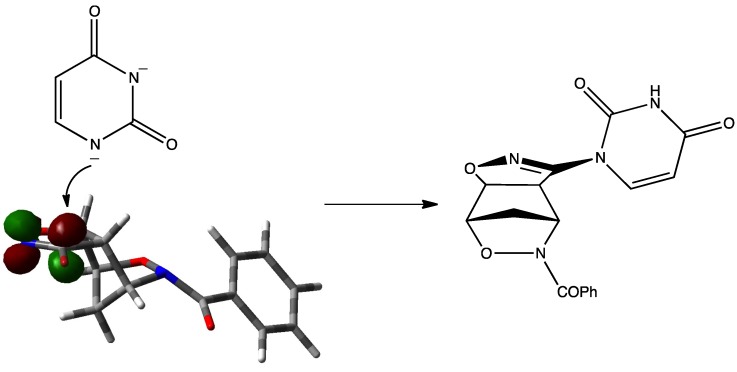
Nucleophile addition orientation.

The presence of a nitrogen atom, belonging to the isoxazoline moiety, in the α position with respect to the =C-Br sp^2^ carbon centre can suggest the A-ER as the operating mechanism. The nitrogen atom helps the addition step allowing to stabilize the negative charge generated in the intermediate that however suffers somewhat of the increased steric requirements determined by the hybridation change of the C-Br carbon atom (Scheme 5) [26].

We believe that stereochemical requirements and nucleophile concentration have a consequence in the experimental conditions we have previously detailed. For this reason the generation under vacuum of the anion of the uracil bases can be considered a success from the experimental point of view. Temperature is also important; lower temperatures do not allowed for obtaining the products in good yields while higher temperature resulted in the decomposition of the reagents.

As final remark, we wish to point out that we have detailed the methodology to promote a high yield process to substitute the bromine in regioisomeric bromoisoxazoline derivatives to insert pyrimidine heterobases on a three-dimensional heterocyclic system. The protocol has to be adapted and properly tuned for the purine-type heterobases, which suffer from the known possibility to give regioisomeric adducts at the N7 and N9 nitrogen atoms. Preliminary results indicate that mixtures of compounds are obtained although in lower yields and for this reason some changes in the method had to be done to make the protocol of general application.

## 3. Experimental

### 3.1. General Information

All melting points are uncorrected. Elemental analyses were done on a C. Erba 1106 elemental analyzer available in our Department. IR spectra (nujol mulls) were recorded on an FT-IR Perkin-Elmer RX-1. ^1^H- and ^13^C-NMR spectra were recorded on a Bruker AVANCE 300 in DMSO-*d*_6_. Chemical shifts are expressed in ppm (δ) from internal tetramethylsilane. Column chromatography: silica gel 60 (0.063–0.200 mm; Merck, Darmstadt, Germany); eluant was a cyclohexane/ethyl acetate gradient from 9:1 to 5:5. For MPLC a Biotage FMP apparatus equipped with KP-SIL columns, eluant cyclohexane/ethyl acetate from 9:1 to 7:3 was used. TLC was performed on Merck silica gel F254 plates. The identification of samples from different experiments was secured by mixed mps and superimposable IR spectra. Glyoxylic acid (Sigma-Aldrich, St. Louis, MO, USA) was used to prepare the bromoxime **2** according to the reported method [15]. *N*-Benzoyl-2-oxa-3-azanorborn-5-ene (**1**) was prepared according to the reported method [1,2,3,4,5,6,7,8,9,10]. Anhydrous DMF was purchased from Sigma-Aldrich. Other chemicals and solvents were purchased from Sigma-Aldrich and Carlo Erba Reagenti (Rodano - MI, Italy) and used without any further purification.

### 3.2. Cycloaddition of Bromonitrile Oxide **3** to N-Benzoyl-2,3-oxazanorborn-5-ene (**1**)

To a stirred solution of *N*-benzoyl-2,3-oxazanorborn-5-ene (**1**, 5.28 g, 26.24 mmol) in EtOAc (200 mL) NaHCO_3_ (3.93 g, 47.2 mmol) was added. To this suspension bromoxime **2** (7.98 g, 39.4 mmol) dissolved in EtOAc (50 mL) were added dropwise. The reaction proceeded under stirring for 48 h at room temperature. Upon filtration and evaporation of the solvent, an oily residue is obtained and submitted to chromatographic separation, allowing for the isolation of the regioisomeric cycloadducts **4a**,**b**. The compounds were found to be identical to previously prepared authentic samples [1].

### 3.3. General Procedure for the Synthesis of Compounds **5a**,**b**, **6a**,**b** (**A**–**D**) and **7a**,**b**

In a dry Schlenk tube an excess of 95% NaH (2.1 equivalents) is suspended in anhydrous DMF (20 mg/mL) and vacuum is applied to get rid of the air inside the suspension. The dry uracil bases are added portionwise and vacuum is applied after every addition keeping watch on the gas evolution. The suspension is left under stirring at room temperature for 30 min to complete the nucleophile generation. Finally, 0.8 equivalents of the regioisomeric cycloadducts **4a**,**b** were added in one portion and again vacuum applied. The mixtures were heated at 95 °C under vigorous stirring for 24 h. After this period of time, the reactions were quenched by pouring the solution in the minimum amount of ice (50 g/0.8 equivs. of **4a**,**b**), adjusting the pH to 7 by means of solid NH_4_Cl/NaCl 3:1 and the water phase extracted with dichloromethane (DCM, 3 × 50 mL). The desired adducts **5a**,**b**, **6a**,**b** **(A**–**D)** and **7a**,**b** were purified by column chromatography under medium pressure (MPLC).

*1-(5-Benzoyl-4,5,7,7a-tetrahydro-3aH-4,7-methanoisoxazolo[4,5-d][1,2]oxazin-3-yl)pyrimidine-2,4(1H,3H)-dione* (**5a**): 0.35 g (64%), m.p. 225 °C (dec.), colorless crystals from diisopropyl ether/ethanol. IR: ν_NH_ 3248 cm^−1^, ν_C=O_ 1731 cm^−1^, ν_C=N_ 1694 cm^−1^. ^1^H-NMR (δ): 3.47 (m, 2H, CH_2_), 4.55 (d, 1H *J* = 8 Hz, H_4isox_), 5.06 (s, 1H, CH-N), 5.13 (d, 1H, *J* = 8 Hz, H_5isox_), 5.31 (s, 1H, HC-O), 5.79 (d, 1H, *J* = 8 Hz, =CH-C=O), 7.44 (m, 2H, arom.) 7.57 (m, 1H, arom.), 7.69 (m, 2H, arom.). 7.83 (d, 1H, *J* = 8 Hz, N-CH=), 11.70 (s, 1H, NH). ^13^C-NMR (δ): 30.7, 32.8, 53.8, 79.6, 84.6, 103.9, 128.3, 128.6, 131.9, 132.7, 141.6, 148.8, 153.2, 163.1, 169.9. Elemental analysis: cald. for C_17_H_14_N_4_O_5_ (MW = 354.32) C 57.63, H 3.98, N 15.81. Found C C 57.60, H 3.97, N 15.80.

*1-(6-Benzoyl-4,6,7,7a-tetrahydro-3aH-4,7-methanoisoxazolo[5,4-d][1,2]oxazin-3-yl)pyrimidine-2,4(1H,3H)-dione* (**5b**): 0.27 g (50%), m.p. > 240 °C (dec.), colorless crystals from diisopropyl ether/ethanol. IR: ν_NH_ 3200 cm^−1^, ν_C=O_ 1716 cm^−1^, ν_C=N_ 1646 cm^−1^. ^1^H-NMR (δ): 2.04 (m, 2H, CH_2_), 4.39 (d, 1H *J* = 8 Hz, H_4isox_), 5.00 (s, 1H, CH-N), 5.28 (m, 1H + 1H, H_5isox_ and HC-O), 5.79 (d, 1H, *J* 8 Hz, =CH-C=O), 7.49 (m, 2H, arom.), 7.57 (m, 1H, arom.), 7.69 (m, 2H, arom.), 7.85 (d, 1H, *J* = 8 Hz, N-CH=), 11.72 (s, 1H, NH). ^13^C-NMR (δ): 32.6, 55.0, 60.9, 61.2, 80.1, 83.9, 103.9, 121.4, 128.3, 128.5, 131.8, 133.0, 141.2, 141.5, 148.8, 152.9, 163.0. Elemental analysis: cald. for C_17_H_14_N_4_O_5_ (MW = 354.32) C 57.63, H 3.98, N 15.81. Found C C 57.64, H 4.00, N 15.81.

*1-(5-Benzoyl-4,5,7,7a-tetrahydro-3aH-4,7-methanoisoxazolo[4,5-d][1,2]oxazin-3-yl)-5-fluoropyrimidine-2,4(1H,3H)-dione* (**6aA**): 0.27 g (46%), m.p. 193–198 °C, colorless crystals from diisopropyl ether/ethanol. IR: ν_NH_ 3394 cm^−1^, ν_C=O_ 1733 cm^−1^, ν_C=N_ 1654 cm^−1^. ^1^H-NMR (δ): 1.97 (m, 2H, CH_2_), 4.57 (d, 1H *J* = 8 Hz, H_4isox_), 5.07 (s, 1H, CH-N), 5.15 (d, 1H, *J* = 8 Hz, H_5isox_), 5.34 (s, 1H, HC-O), 7.47 (m, 2H, arom.), 7.57 (m, 1H, arom.), 7.69 (m, 2H, arom.), 8.24 (d, 1H, *J* = 7 Hz, N-CH=), 12.22 (s, 1H, NH). ^13^C-NMR (δ): 32.7, 39.8, 53.6, 79.6, 84.7, 90.0, 127.4, 128.2, 128.3, 128.7, 131.2, 131.8, 132.8, 147.6, 152.9, 157.4, 170.0. Elemental analysis: cald. for C_17_H_13_FN_4_O_5_ (MW = 372.31) C 54.84, H 3.52, N 15.05. Found C 54.85, H 3.50, N 15.06.

*1-(6-Benzoyl-4,6,7,7a-tetrahydro-3aH-4,7-methanoisoxazolo[5,4-d][1,2]oxazin-3-yl)-5-fluoropyrimidine-2,4(1H,3H)-dione* (**6bA**): 0.37 g (64%), m.p. 95–100 °C, colorless crystals from diisopropyl ether/ethanol. IR: ν_NH_ 3157 cm^−1^, ν_C=O_ 1723 cm^−1^, ν_C=N_ 1648 cm^−1^. ^1^H-NMR (δ): 2.04 (s, 2H, CH_2_), 4.39 (d, 1H *J* = 8 Hz, H_4isox_), 5.00 (s, 1H, CH-N), 5.28 (m, ^1^H + ^1^H, H_5isox_ and HC-O), 7.47 (m, 2H, arom.), 7.56 (m, 1H, arom.), 7.71 (m, 2H, arom.), 8.24 (d, 1H, *J* = 7 Hz, N-CH=), 12.25 (s, 1H, NH). ^13^C-NMR (δ): 32.6, 54.8, 56.6, 80.0, 84.0, 125.8, 127.0, 127.4, 128.2, 128.3, 128.5, 131.2, 131.8, 133.0, 147.6, 152.5, 168.9. Elemental analysis: cald. for C_17_H_13_FN_4_O_5_ (MW = 372.31) C 54.84, H 3.52, N 15.05. Found C 54.83, H 3.51, N 15.02.

*1-(5-Benzoyl-4,5,7,7a-tetrahydro-3aH-4,7-methanoisoxazolo[4,5-d][1,2]oxazin-3-yl)-5-chloropyrimidine-2,4(1H,3H)-dione* (**6aB**): 0.32 g (53%), m.p. 189–196 °C, colorless crystals from diisopropyl ether/ethanol. IR: ν_NH_ 3360 cm^−1^, ν_C=O_ 1734 cm^−1^, ν_C=N_ 1703 cm^−1^. ^1^H-NMR (δ): 1.96 (m, 2H, CH_2_), 4.55 (d, 1H *J* = 8 Hz, H_4isox_), 5.08 (s, 1H, CH-N), 5.17 (d, 1H, *J* = 8 Hz, H_5isox_), 5.36 ( s, 1H, HC-O), 7.45 (m, 2H, arom.), 7.57 (m, 1H, arom.), 7.73 (m, 2H, arom.), 8.23 (s, 1H, N-CH=), 12.23 (s, 1H, NH). ^13^C-NMR (δ): 32.8, 41.2, 53.4, 56.3, 84.8, 109.8, 127.4, 128.2, 128.7, 131.9, 132.7, 138.7, 148.2, 159.0, 172.4. Elemental analysis: cald. for C_17_H_13_ClN_4_O_5_ (MW = 388.76) C 52.52, H 3.37, N 14.41. Found C 52.54, H 3.35, N 14.40.

*1-(6-Benzoyl-4,6,7,7a-tetrahydro-3aH-4,7-methanoisoxazolo[5,4-d][1,2]oxazin-3-yl)-5-chloropyrimidine-2,4(1H,3H)-dione* (**6bB**): 0.46 g (76%), m.p. 98–105 °C, colorless crystals from diisopropyl ether/ethanol. IR: ν_NH_ 3178 cm^−1^, ν_C=O_ 1732 cm^−1^, ν_C=N_ 1708 cm^−1^. ^1^H-NMR (δ): 2.07 (m, 2H, CH_2_), 4.38 (d, 1H *J* = 9 Hz, H_4isox_), 5.00 (s, 1H, CH-N), 5.28 ( s, 1H, HC-O), 5.32 (d, 1H, *J* = 9 Hz, H_5isox_), 7.46 (m, 2H, arom.), 7.58 (m, 1H, arom.), 7.70 (m, 2H, arom.), 8.24 (s, 1H, N-CH=), 12.25 (s, 1H, NH). ^13^C-NMR (δ): 32.7, 35.8, 54.6, 79.8, 84.1, 109.8, 127.4, 128.2, 128.3, 128.5, 131.8, 133.0, 138.6, 148.2, 152.5, 159.0, 162.3, 168.9. Elemental analysis: cald. for C_17_H_13_ClN_4_O_5_ (MW = 388.76) C 52.52, H 3.37, N 14.41. Found C 52.50, H 3.36, N 14.42.

*1-(5-Benzoyl-4,5,7,7a-tetrahydro-3aH-4,7-methanoisoxazolo[4,5-d][1,2]oxazin-3-yl)-5-bromopyrimidine-2,4(1H,3H)-dione* (**6aC**): 0.38 g (56%), m.p. 96–100 °C, colorless crystals from diisopropyl ether/ethanol. IR: ν_NH_ 3200 cm^−1^, ν_C=O_ 1734 cm^−1^, ν_C=N_ 1621 cm^−1^. ^1^H-NMR (δ): 1.95 (m, 2H, CH_2_), 4.54 (d, 1H *J* = 10 Hz, H_4isox_), 5.08 (s, 1H, CH-N), 5.16 (d, 1H, *J* = 10 Hz, H_5isox_), 5.36 (s, 1H, HC-O), 7.47 (m, 2H, arom.), 7.57 (m, 1H, arom.), 7.68 (m, 2H, arom.), 8.27 (s, 1H, N-CH=), 12.18 (s, 1H, NH). ^13^C-NMR (δ): 30.7, 32.8, 53.4, 79.6, 84.8, 98.6, 128.2, 128.7, 131.8, 132.8, 140.9, 148.4, 152.7, 159.2. Elemental analysis: cald. for C_17_H_13_BrN_4_O_5_ (MW = 433.21) C 47.13, H 3.02, N 12.93. Found C 47.10, H 3.01, N 12.95.

*1-(6-Benzoyl-4,6,7,7a-tetrahydro-3aH-4,7-methanoisoxazolo[5,4-d][1,2]oxazin-3-yl)-5-bromopyrimidine-2,4(1H,3H)-dione* (**6bC**): 0.33 g (49%), m.p. 104–106 °C, colorless crystals from diisopropyl ether/ethanol. IR: ν_NH_ 3121 cm^−1^, ν_C=O_ 1732 cm^−1^, ν_C=N_ 1702 cm^−1^. ^1^H-NMR (δ): 2.07 (m, 2H, CH_2_), 4.37 (d, 1H *J* = 9 Hz, H_4isox_), 5.00 (s, 1H, CH-N), 5.28 (s, 1H, HC-O), 5.31 (d, 1H, *J* = 9 Hz, H_5isox_), 7.49 (m, 2H, arom.), 7.57 (m, 1H, arom.), 7.69 (m, 2H, arom.), 8.29 (s, 1H, N-CH=), 12.20 (s, 1H, NH). ^13^C-NMR (δ): 26.3, 32.7, 54.6, 79.8, 84.1, 98.6, 128.3, 128.5, 131.7, 132.4, 133.0, 140.9, 148.4, 152.4, 159.1, 165.7, 168.9. Elemental analysis: cald. for C_17_H_13_BrN_4_O_5_ (MW = 433.21) C 47.13, H 3.02, N 12.93. Found C 47.14, H 3.03, N 12.95.

*1-(5-Benzoyl-4,5,7,7a-tetrahydro-3aH-4,7-methanoisoxazolo[4,5-d][1,2]oxazin-3-yl)-5-iodopyrimidine-2,4(1H,3H)-dione* (**6aD**): 0.42 g (57%), m.p. 110–115 °C, colorless crystals from diisopropyl ether/ethanol. IR: ν_NH_ 3394 cm^−1^, ν_C=O_ 1732 cm^−1^, ν_C=N_ 1654 cm^−1^. ^1^H-NMR (δ): 1.95 (m, 2H, CH_2_), 4.53 (d, 1H *J* = 9 Hz, H_4__isox_), 5.07 (s, 1H, CH-N), 5.15 (d, 1H, *J* = 9 Hz, H_5 isox_), 5.34 (s, 1H, HC-O), 7.46 (m, 2H, arom.), 7.57 (m, 1H, arom.), 7.67 (m, 2H, arom.), 8.23 (s, 1H, N-CH=), 12.04 (s, 1H, NH). ^13^C-NMR (δ): 26.3, 32.8, 53.4, 73.0, 79.6, 74.7, 127.4 128.2, 128.6, 128.7, 131.8, 132.8, 145.3, 148.8, 152.6, 166.0. Elemental analysis: cald. for C_17_H_13_IN_4_O_5_ (MW = 480.21) C 42.52, H 2.73, N 11.67. Found C 42.50, H 2.75, N 11.66.

*1-(6-Benzoyl-4,6,7,7a-tetrahydro-3aH-4,7-methanoisoxazolo[5,4-d][1,2]oxazin-3-yl)-5-iodopyrimidine-2,4(1H,3H)-dione* (**6bD**): 0.42 g (57%), m.p. 162–164 °C, colorless crystals from diisopropyl ether/ethanol. IR: ν_NH_ 3160 cm^−1^, ν_C=O_ 1720 cm^−1^, ν_C=N_ 1654 cm^−1^. ^1^H-NMR (δ): 2.05 (m, 2H, CH_2_), 4.35 (d, 1H *J* = 9 Hz, H_4isox_), 4.98 (s, 1H, CH-N), 5.26 ( s, 1H, HC-O), 5.28 (d, 1H, *J* = 9 Hz, H_5isox_), 7.52 (m, 3H, arom.), 7.68 (m, 2H, arom.), 8.24 (s, 1H, N-CH=), 12.05 (s, 1H, NH). ^13^C-NMR (δ): 32.7, 39.8, 54.7, 73.0, 79.9, 84.2, 127.4, 128.3, 128.4, 129.2, 131.7, 132.7, 142.1, 145.3, 148.8, 152.4, 160.7. Elemental analysis: cald. for C_17_H_13_IN_4_O_5_ (MW = 480.21) C 42.52, H 2.73, N 11.67. Found C 42.51, H 2.76, N 11.68.

*1-(5-Benzoyl-4,5,7,7a-tetrahydro-3aH-4,7-methanoisoxazolo[4,5-d][1,2]oxazin-3-yl)-5-methylpyrimidine-2,4(1H,3H)-dione* (**7a**): 0.26 g (45%), m.p. 215–217 °C, colorless crystals from diisopropyl ether/ethanol. IR: ν_NH_ 3176 cm^−1^, ν_C=O_ 1715 cm^−1^, ν_C=N_ 1674 cm^−1^. ^1^H-NMR (δ): 1.81 (d, 3H, *J* = 1 Hz, CH_3_), 1.92 (s, 2H, CH_2_), 4.58 (d, 1H *J* = 8 Hz, H_4isox_), 5.07 (s, 1H, CH-N), 5.12 (d, 1H, *J* = 8 Hz, H_5isox_), 5.30 (s, 1H, HC-O), 7.51 (m, 3H, arom.), 7.69 (d, 1H, *J* = 1 Hz, N-CH=), 7.73 (m, 2H, arom.), 11.70 (s, 1H, NH). ^13^C-NMR (δ): 11.9, 32.9, 54.1, 60.1, 79.8, 84.6, 111.8, 128.3, 128.8, 131.9, 133.1, 137.0, 148.9, 153.3, 163.8, 170.0. Elemental analysis: cald. for C_18_H_16_N_4_O_5_ (MW = 368.34) C 58.69, H 4.38, N 15.21. Found C 58.70, H 4.39, N 15.19.

*1-(6-Benzoyl-4,6,7,7a-tetrahydro-3aH-4,7-methanoisoxazolo[5,4-d][1,2]oxazin-3-yl)-5-methylpyrimidine-2,4(1H,3H)-dione* (**7b**): 0.31 g (55%), m.p. 220–222 °C, colorless crystals from diisopropyl ether/ethanol. IR: ν_NH_ 3149 cm^−1^, ν_C=O_ 1710 cm^−1^, ν_C=N_ 1698 cm^−1^. ^1^H-NMR (δ): 1.82 (d, 3H, *J* = 1 Hz, CH_3_), 2.04 (m, 2H, CH_2_), 4.41 (d, 1H *J* = 8 Hz, H_4isox_), 4.99 (s, 1H, CH-N), 5.25 (d, 1H, *J* = 8 Hz, H_5isox_), 5.27 (s, 1H, HC-O), 7.51 (m, 3H, arom.), 7.69 (d, 1H, *J* = 1 Hz, N-CH=), 7.73 (m, 2H, arom.), 11.63 (s, 1H, NH). ^13^C-NMR (δ): 11.9, 32.6, 55.1, 61.2, 80.1, 83.7, 111.7, 128.3, 128.5, 131.8, 130.0, 136.8, 148.8, 152.9, 163.7, 168.9. Elemental analysis: cald. for C_18_H_16_N_4_O_5_ (MW = 368.34) C 58.69, H 4.38, N 15.21. Found C 58.71, H 4.37, N 15.20.

### 3.4. X-ray Crystallographic Analysis of Compounds **5a** and **7a**

Unit cell dimensions for compounds **5a** and **7a** were obtained by least-squares fitting of 2θ values for 25 reflections, with an Enraf–Nonius CAD4 diffractometer and graphite-monochromated Mo–*K*α radiation at the Centro Grandi Strumenti (CGS) of the University of Pavia, Italy. The structure was solved by direct methods, and the E-map correctly revealed the non-hydrogen atoms in the molecules. The positions of the hydrogen atoms were located by difference Fourier synthesis, compared with those calculated from the geometry of the molecules and refined isotropically in the subsequent least-squares refinement. The program SHELXL [27] was used to solve the structure. The ORTEP program [28] was used for molecular graphics. The ORTEP plots of 8-benzoyl-5-uracil-3,9-dioxa-4,8-diaza-[5.2.1.0^2.5^]-dec-4-ene (**5a**) and 8-benzoyl-5-thymine-3,9-dioxa-4,8-diaza-[5.2.1.0^2.5^]dec-4-ene (**7a**) are shown in Figure 1 and Figure 2 and the corresponding bond lengths and angles are given in Table 2.

A total 2,893 reflections for **5a** and 1,828 for **7a** was explored with the ω/2θ scan tecnique. Correction was applied for Lorentz and polarization. An approximate scale and a mean thermal factor of 2.867 Å^2^ and 3.417 Å^2^ were determined by Wilson statistic [29] for **5a** and **7a**. The structure was solved by direct methods and *E*-map correctly revealed all the non-hydrogen atoms in the molecules. The position of the hydrogen atoms was checked in a final difference Fourier-map and refined isotropically in the subsequent least- squares refinement. The experimental crystal X-ray data are summarized in Table 3 and Table 4, respectively. CCDC deposition numbers: (**5a**) 987413 and (**7a**) 987414.

**Table 2:** Selected bond lengths (Å) and angles (deg) (with Esd’s in Parentheses). Compounds **5a** **7a** **5a** **7a**.

C1—C2	1.523(4) 1.507(6)	C5—C6	1.499(3) 1.498(5)
C1—O9	1.458(3) 1.456(5)	C5—N11	1.417(3) 1.408(4)
C1—C10	1.501(4) 1.497(6)	C6—C7	1.538(4) 1.533(5)
C2—O3	1.452(3) 1.453(4)	C7—N8	1.477(3) 1.487(5)
C2—C6	1.534(3) 1.540(5)	C7—C10	1.517(3) 1.520(6)
O3—N4	1.420(2) 1.402(4)	N8—O9	1.450(2) 1.455(4)
N4—C5	1.270(3) 1.272(4)	N8—C18	1.376(3) 1.377(5)
Bond Angles (°)			
C2—C1—O9	104.8(2) 104.3(2)	C2—C6—C5	100.1(2) 99.8(3)
C2—C1—C10	103.9(2) 104.2(4)	C2—C6—C7	101.9(2) 101.7(3)
O9—C1—C10	103.3(2) 103.8(4)	C6—C7—N8	104.0(2) 104.2(3)
C1—C2—C6	102.6(2) 102.9(3)	C6—C7—C10	103.2(2) 102.7(4)
O3—C2—C6	105.8(2) 105.5(3)	N8—C7—C10	101.6(2) 101.2(3)
C2—O3—N4	109.4(2) 109.4(3)	C7—N8—O9	105.2(2) 105.3(3)
O3—N4—C5	109.1(2) 109.9(3)	C7—N8—C18	126.9(2) 124.1(3)
N4—C5—C6	115.6(2) 115.1(3)	O9—N8—C18	112.9(2) 110.0(3)
N4—C5—N11	118.1(2) 116.4(3)	C1—O9—N8	103.7(2) 103.4(3)
C6—C5—N11	126.4(2) 128.3(3)	C1—C10—C7	92.7(2) 92.9(4)

**Table 3:** Crystal data, data collection and structure refinement (compound **5a**).

Empirical Formula	C_17_H_14_N_4_O_5_
Formula weight	354.32
Crystal size, mm	0.56 × 0.42 × 0.18
Temperature, K	293
Crystal system	Orthorhombic
Space group	*P* bca
*a*, Å	9.977(4)
*b*, Å	16.774(3)
*c*, Å	19.649(3)
α	90
β	90
γ	90
*V*, Å^3^	3288(1)
*Z*	8
*D_calcd_*, g·cm^−3^	1.431
Absorption coeff., µ, mm^−1^	0.108
Diffractometer/scan	Enraf–Nonius CAD–4, θ/2θ
λ, Å	0.71073
*F*(000)	1472
Range (°) for data	2.0 < θ > 25
Index ranges	0 < *h>* 11, 0 < *k>* 19, 0 < *l* > 23
No. of reflects. measd	2893
No. of unique reflects	1824
Correction applied	Lorentz–polarization
Refinement method	Full–matrix least–squares
No. of variables	291
Goodness–of–fit (2893)	0.900
*R*_1_ (*I*) > 2 σ (*I*), (1824)	0.0403
*R*_1_ (2883)	0.0785
(∆ρ) max, min, eÅ^−3^	0.124, −0.151

**Table 4:** Crystal data, data collection and structure refinement (compound **7a**).

Empirical Formula	C_20_H_22_N_4_O_6_
Formula weight	414.417
Crystal size, mm	0.50 × 0.385 × 0.14
Temperature, K	293
Crystal system	Monoclinic
Space group	*P* 2_1_/*n*
*a*, Å	11.991(3)
*b*, Å	11.4437(8)
*c*, Å	14.63(1)
α	90.0
β	101.34(4)
γ	90.0
*V*, Å^3^	1968.3(14)
*Z*	4
*D_calcd_*, g·cm^−3^	1.3985
Absorption coeff., µ, mm^−1^	0.1051
Diffractometer/scan	Enraf–Nonius CAD–4, θ/2 θ
Radiation	MoKα
λ, Å	0.71073
*F*(000)	872
Range (°) for data	2.0 < θ > 25.0
Index ranges	−11 < *h* > 11, 0 < *k* > 10, 0 < *l* > 14
No. of reflects. measd	1828
No. of unique reflects	1266
Correction applied	Lorentz–polarization
Refinement method	Full–matrix least–squares
No. of variables	359
Goodness–of–fit (1828)	1.024
*R*_1_ (*I*) > 2 σ (*I*), (1266)	0.0414
*R*_1_ (1828)	0.0728
(∆ρ) max, min, eÅ^−3^	0.119, −0.129

## 4. Conclusions

The regioisomeric cycloadducts of the bromonitrile oxide to the *N*-benzoyl-2,3-oxazanorborn-5-ene were easily prepared and elaborated into a novel class of uracil-based scaffolds. The key synthetic step is represented by the nucleophilic substitution at the sp^2^ carbon atom of the bromoisoxazoline three-dimensional heterocycles. The protocol to perform the nucleophilic substitution of uracil anions was revised, and optimized as well as adapted to the steric requirements of the substrates.

Regarding the uracil derivatives of type **5**, **6** and **7** the synthetic elaboration will continue with standard hydrolysis for the detachment of the benzoyl group and hydrogenolysis of the resulting N-O bond to give the desired aminols. These latter represent a novel class of nucleoside analogues [21] to be tested against a variety of viruses or used as synthons for the preparation of β-turn inducers containing nucleobases for their insertion in PNAs [12,30,31].

## Supplementary Materials

Supplementary materials can be accessed at: http://www.mdpi.com/1420-3049/19/6/8661/s1.
