# Supplementary material for: Three-Dimensional Heterocycles: New Uracil-Based Structures Obtained by Nucleophilic Substitution at the sp2 Carbon of Bromoisoxazoline
Source: Molecules. 2014 Jun 24;19(6):8661–78. doi: 10.3390/molecules19068661 (PMC6271772; doi:10.3390/molecules19068661)
Supplement: Supplementary file 1 [file molecules-19-08661-s001.pdf]

Supplementary Information

^{1}H and ^{13}C-NMR spectra of the newly reported compounds.

5a.

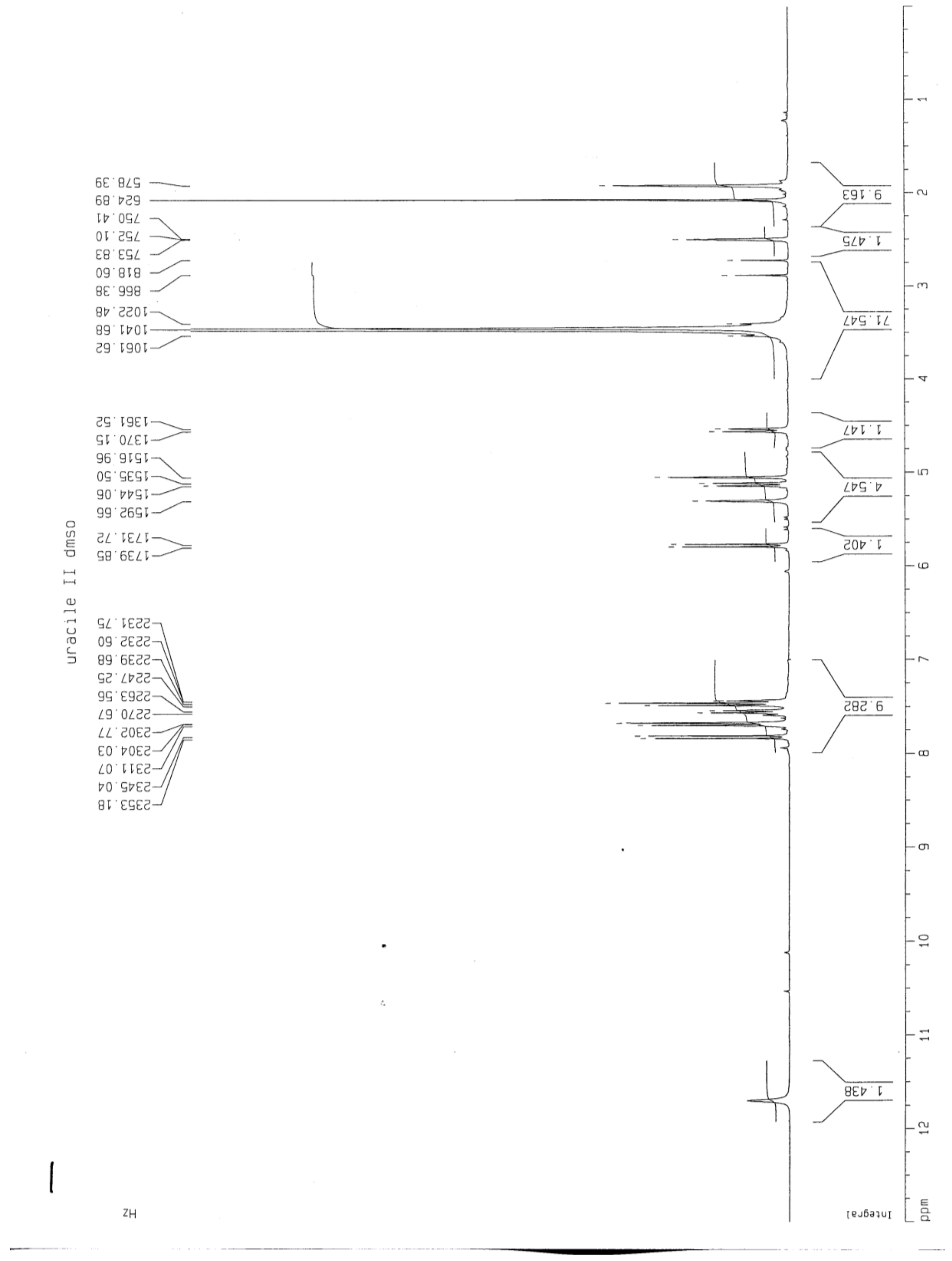

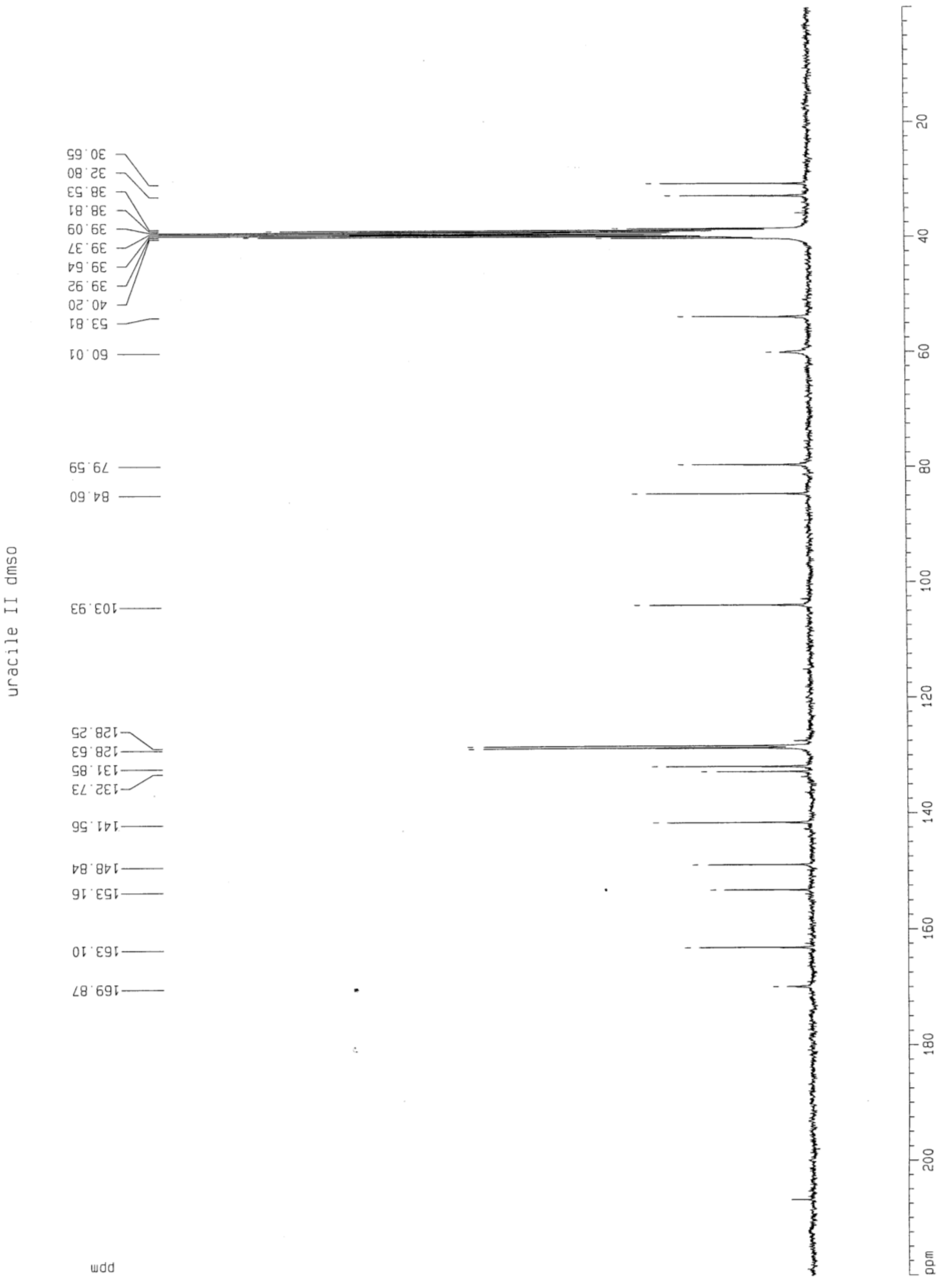
uracile II dmso
30.65
32.80
38.53
38.81
39.09
39.37
39.64
39.92
40.20
53.81
60.01
79.59
84.60
103.93
128.25
128.63
131.85
132.73
141.56
148.84
153.16
163.10
169.87
ppm
20
40
60
80
100
120
140
160
180
200
ppm

5b.

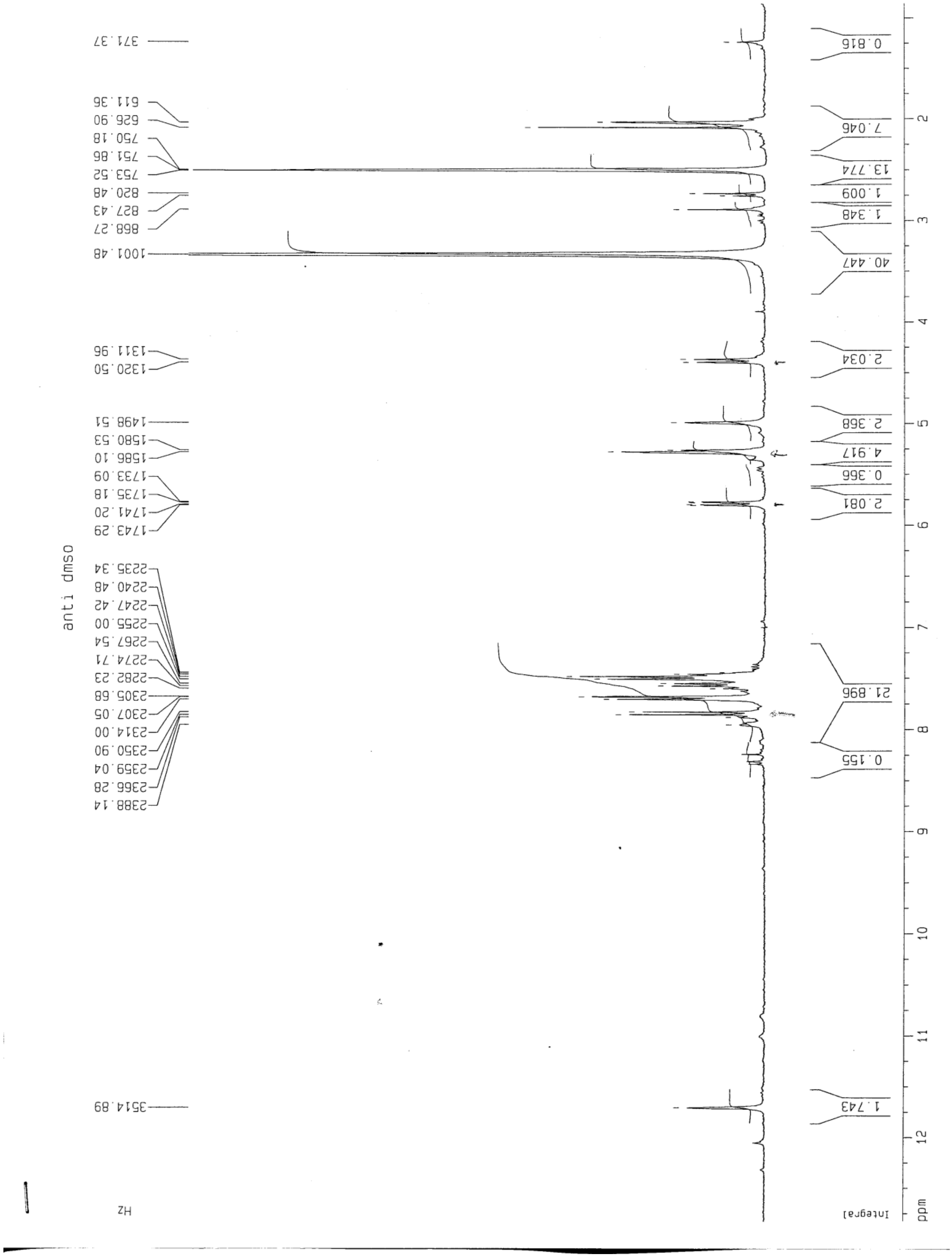
anti dmso
371.37
611.36
626.90
750.18
751.86
753.52
820.48
827.43
868.27
1001.48
1311.96
1320.50
1498.51
1580.53
1586.10
1733.09
1735.18
1741.20
1743.29
2235.34
2240.48
2247.42
2255.00
2267.54
2274.71
2282.23
2305.68
2307.05
2314.00
2350.90
2359.04
2366.28
2388.14
3514.89
Hz
0.816
7.046
13.774
1.009
1.348
40.447
2.034
2.368
4.917
0.366
2.081
21.896
0.155
1.743
Integral
ppm

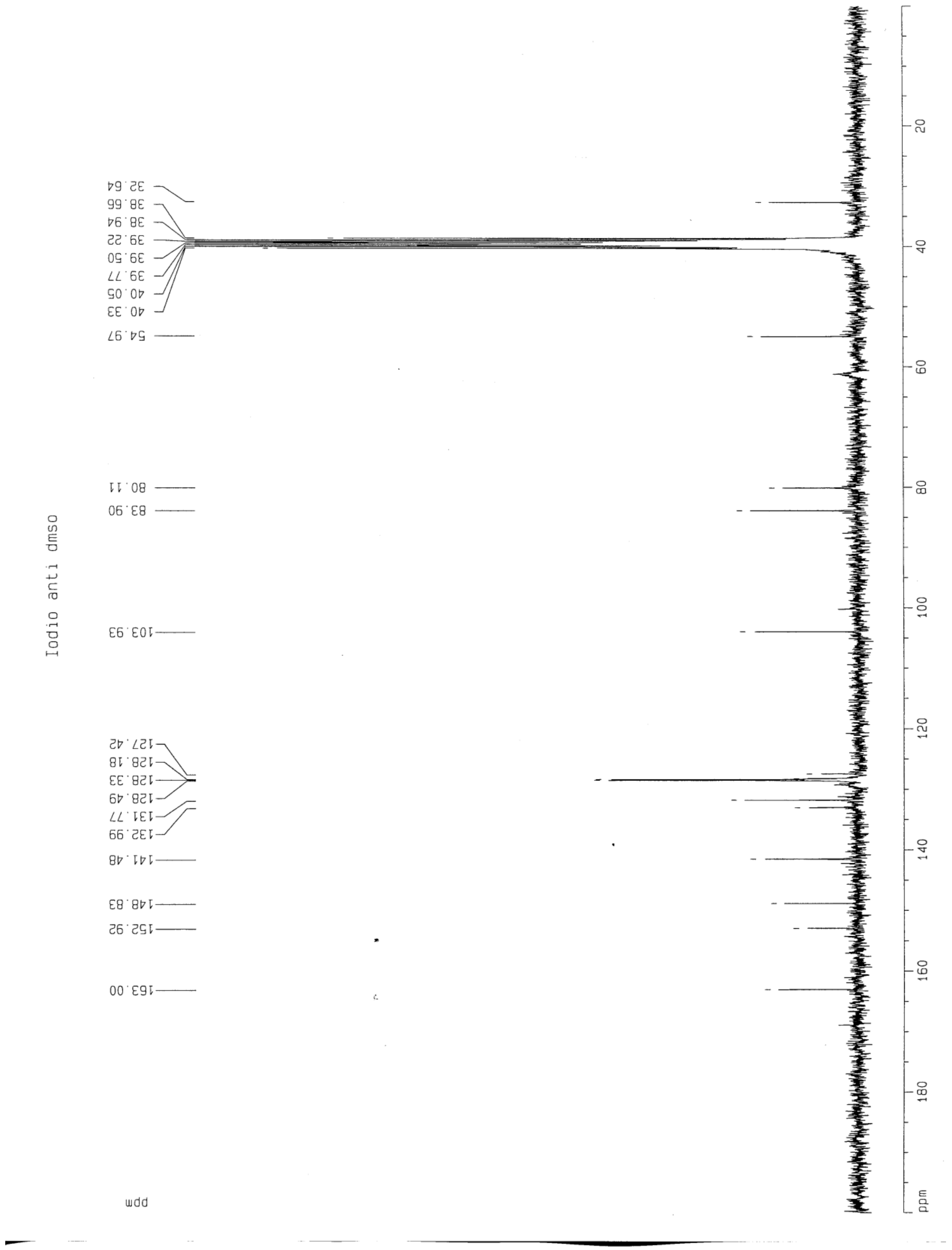
Iodio anti dmso
32.64
38.66
38.94
39.22
39.50
39.77
40.05
40.33
54.97
80.11
83.90
103.93
127.42
128.18
128.33
128.49
131.77
132.99
141.48
148.83
152.92
163.00
ppm
20
40
60
80
100
120
140
160
180
ppm

6aA.

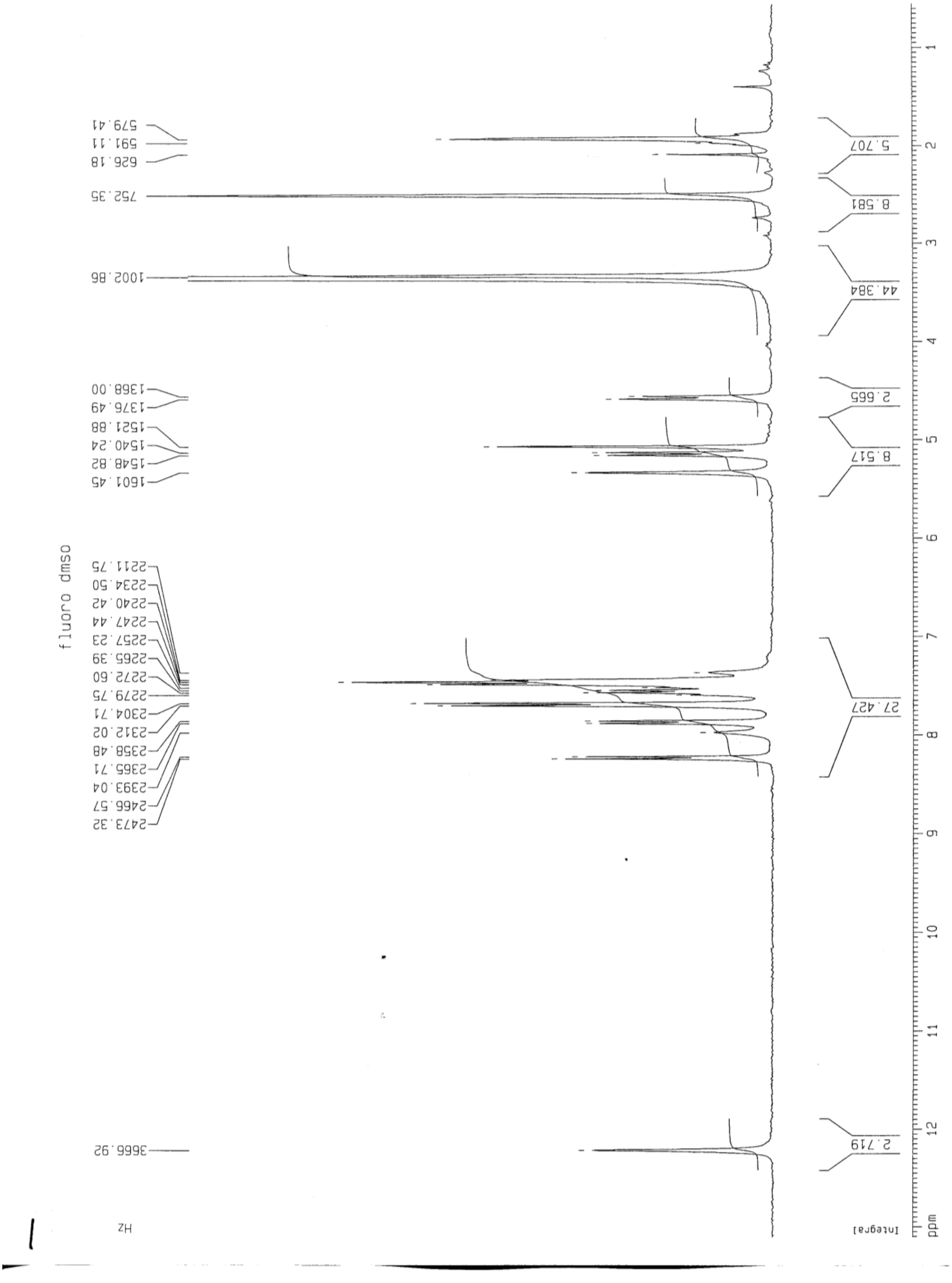
fluoro dmso
579.41
591.11
626.18
752.35
1002.86
1368.00
1376.49
1521.88
1540.24
1548.82
1601.45
2211.75
2234.50
2240.42
2247.44
2257.23
2265.39
2272.60
2279.75
2304.71
2312.02
2358.48
2365.71
2393.04
2466.57
2473.32
3666.92
Hz
5.707
8.581
44.384
2.665
8.517
27.427
2.719
Integral
ppm
1
2
3
4
5
6
7
8
9
10
11
12

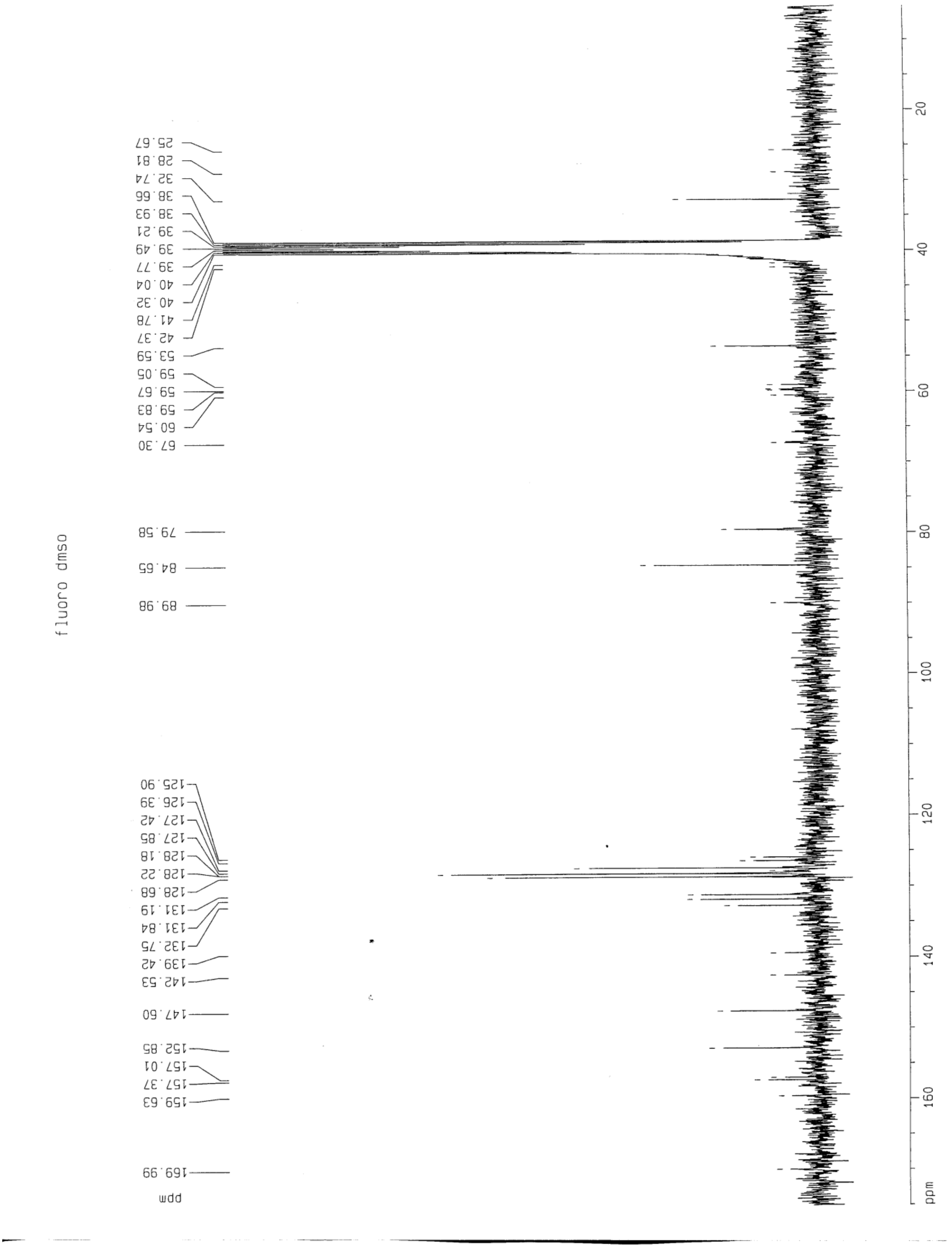
fluoro dmso
25.67
28.81
32.74
38.66
38.93
39.21
39.49
39.77
40.04
40.32
41.78
42.37
53.59
59.05
59.67
59.83
60.54
67.30
79.58
84.65
89.98
125.90
126.39
127.42
127.85
128.18
128.22
128.68
131.19
131.84
132.75
139.42
142.53
147.60
152.85
157.01
157.37
159.63
169.99
ppm
20
40
60
80
100
120
140
160
ppm

6bA.

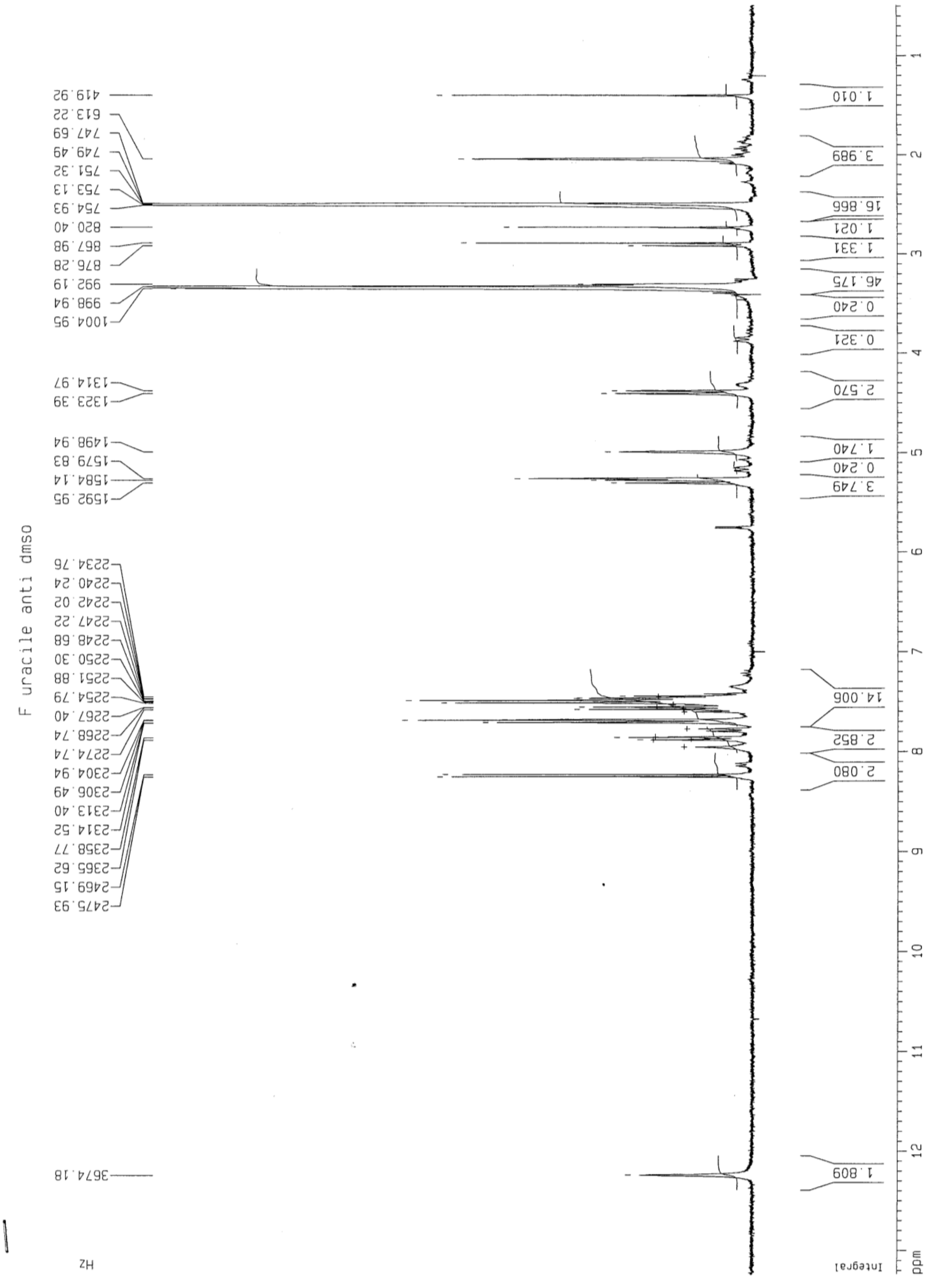

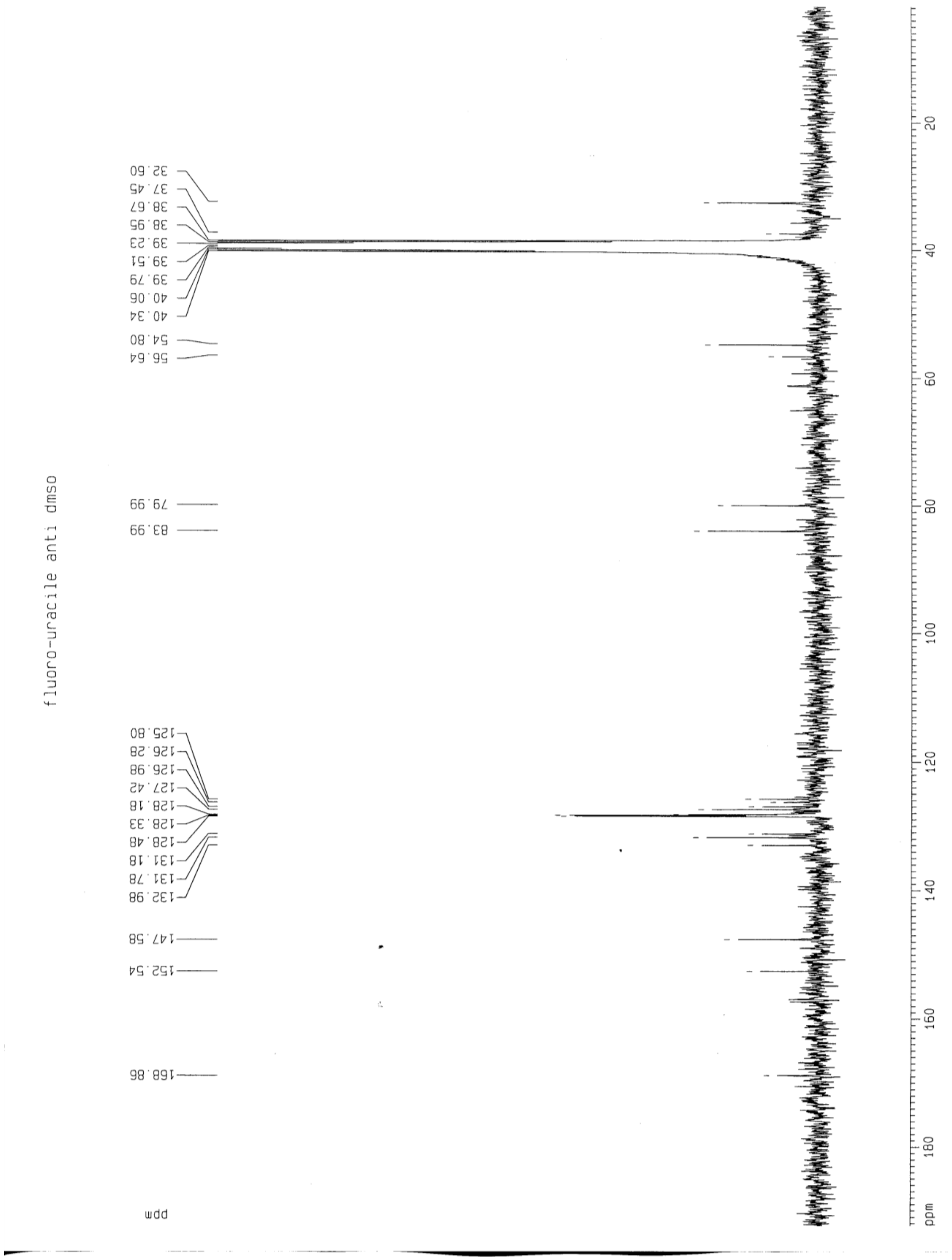
fluoro-uracile anti dmso
32.60
37.45
38.67
38.95
39.23
39.51
39.79
40.06
40.34
54.80
56.64
79.99
83.99
125.80
126.28
126.98
127.42
128.18
128.33
128.48
131.18
131.78
132.98
147.58
152.54
168.86
ppm
20
40
60
80
100
120
140
160
180
ppm

6aB.

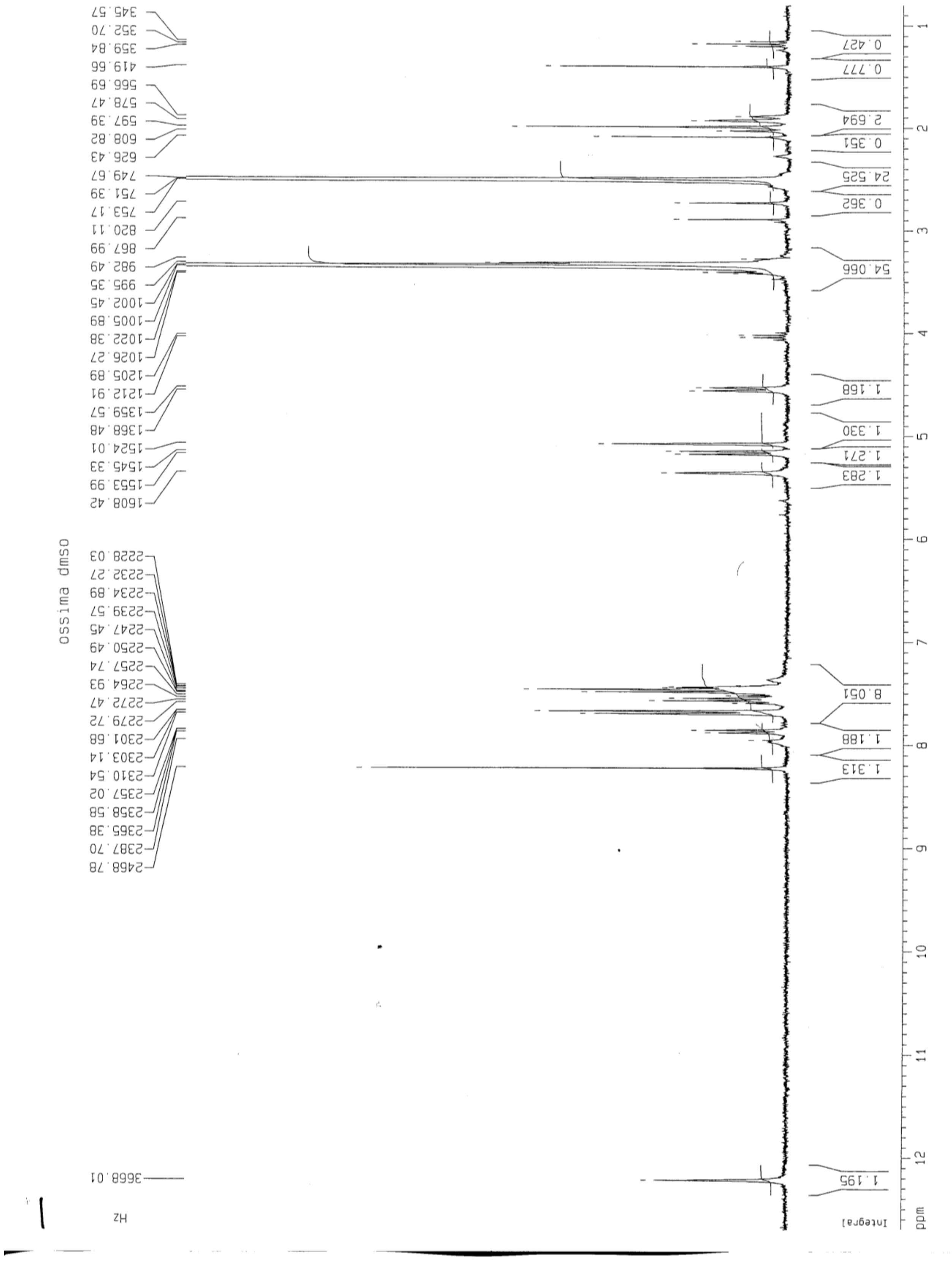
345.57
352.70
359.84
419.66
566.69
578.47
597.39
608.82
626.43
749.67
751.39
753.17
820.11
867.99
982.49
995.35
1002.45
1005.89
1022.38
1026.27
1205.89
1212.91
1359.57
1368.48
1524.01
1545.33
1553.99
1608.42
ossima dmso
2228.03
2232.27
2234.89
2239.57
2247.45
2250.49
2257.74
2264.93
2272.47
2279.72
2301.68
2303.14
2310.54
2357.02
2358.58
2365.38
2387.70
2468.78
3668.01
Hz
0.427
0.777
2.694
0.351
24.525
0.362
54.066
1.168
1.330
1.271
1.283
8.051
1.188
1.313
1.195
Integral
ppm

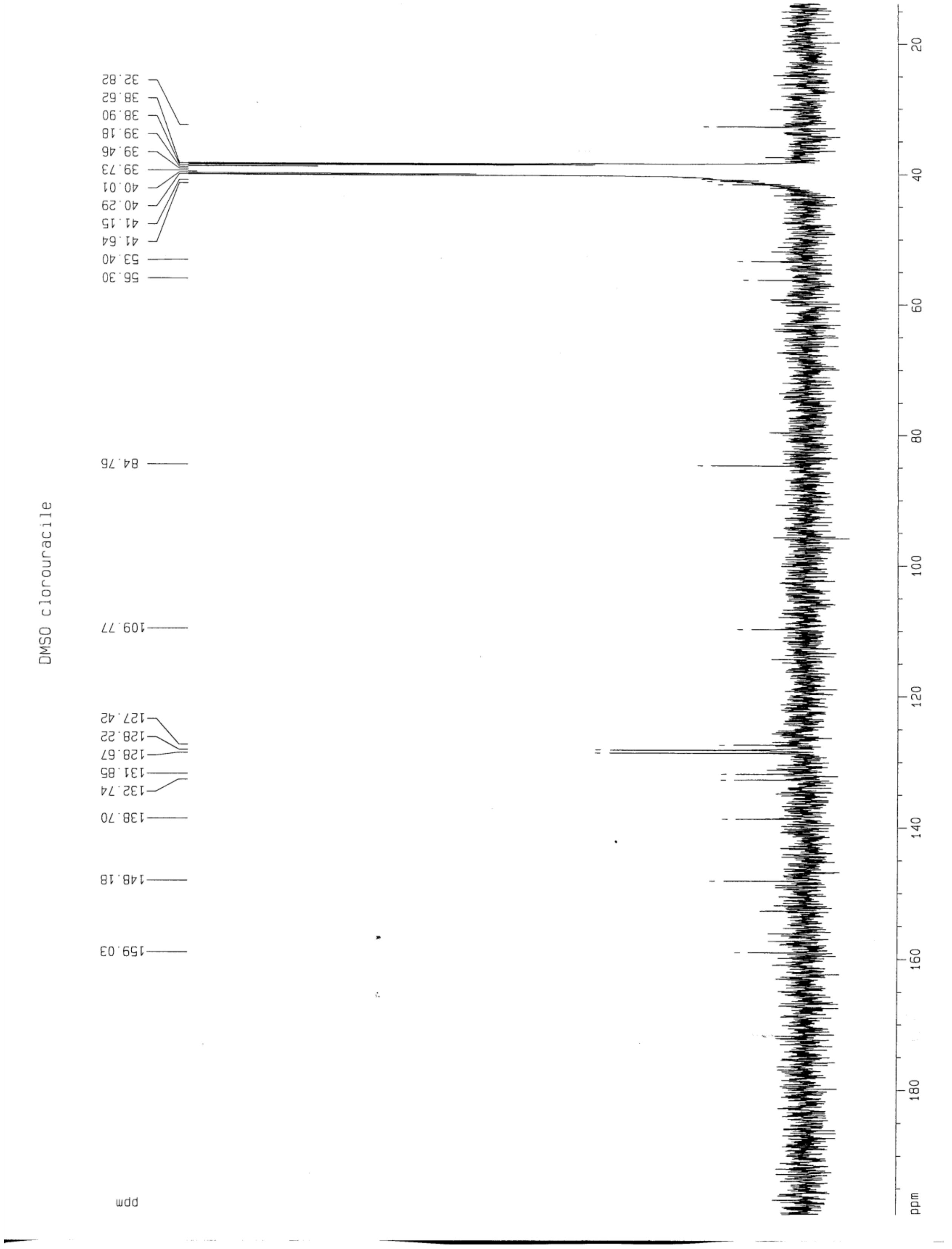
DMSO clorouracile
32.82
38.62
38.90
39.18
39.46
39.73
40.01
40.29
41.15
41.64
53.40
56.30
84.76
109.77
127.42
128.22
128.67
131.85
132.74
138.70
148.18
159.03
ppm
20
40
60
80
100
120
140
160
180
ppm

6bB.

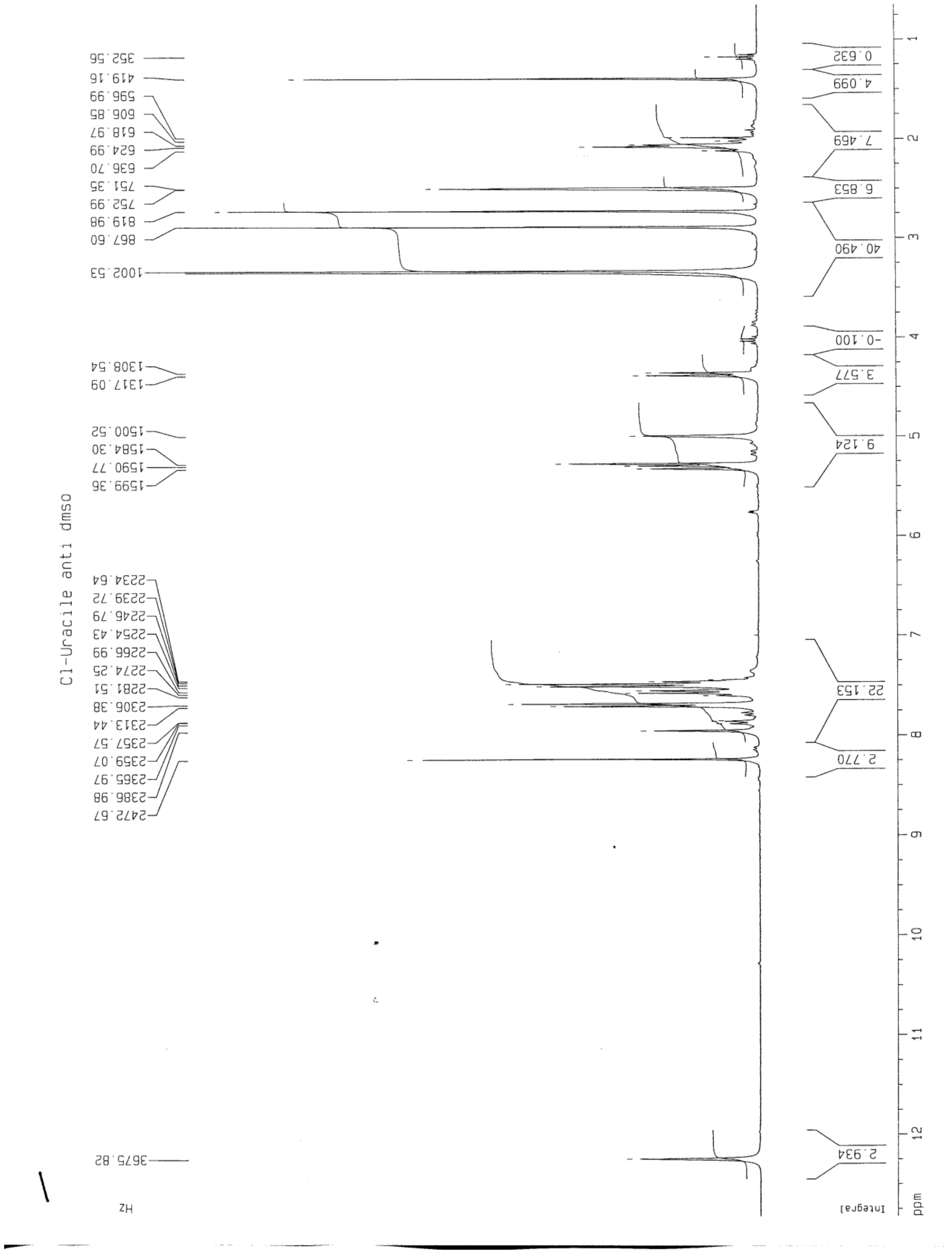
Cl-Uracile anti dmso
352.56
419.16
596.99
606.85
618.97
624.99
636.70
751.35
752.99
819.98
867.60
1002.53
1308.54
1317.09
1500.52
1584.30
1590.77
1599.36
2234.64
2239.72
2246.79
2254.43
2266.99
2274.25
2281.51
2306.38
2313.44
2357.57
2359.07
2365.97
2386.98
2472.67
3675.82
Hz
0.632
4.099
7.469
6.853
40.490
-0.100
3.577
9.124
22.153
2.770
2.934
Integral
1 2 3 4 5 6 7 8 9 10 11 12
ppm

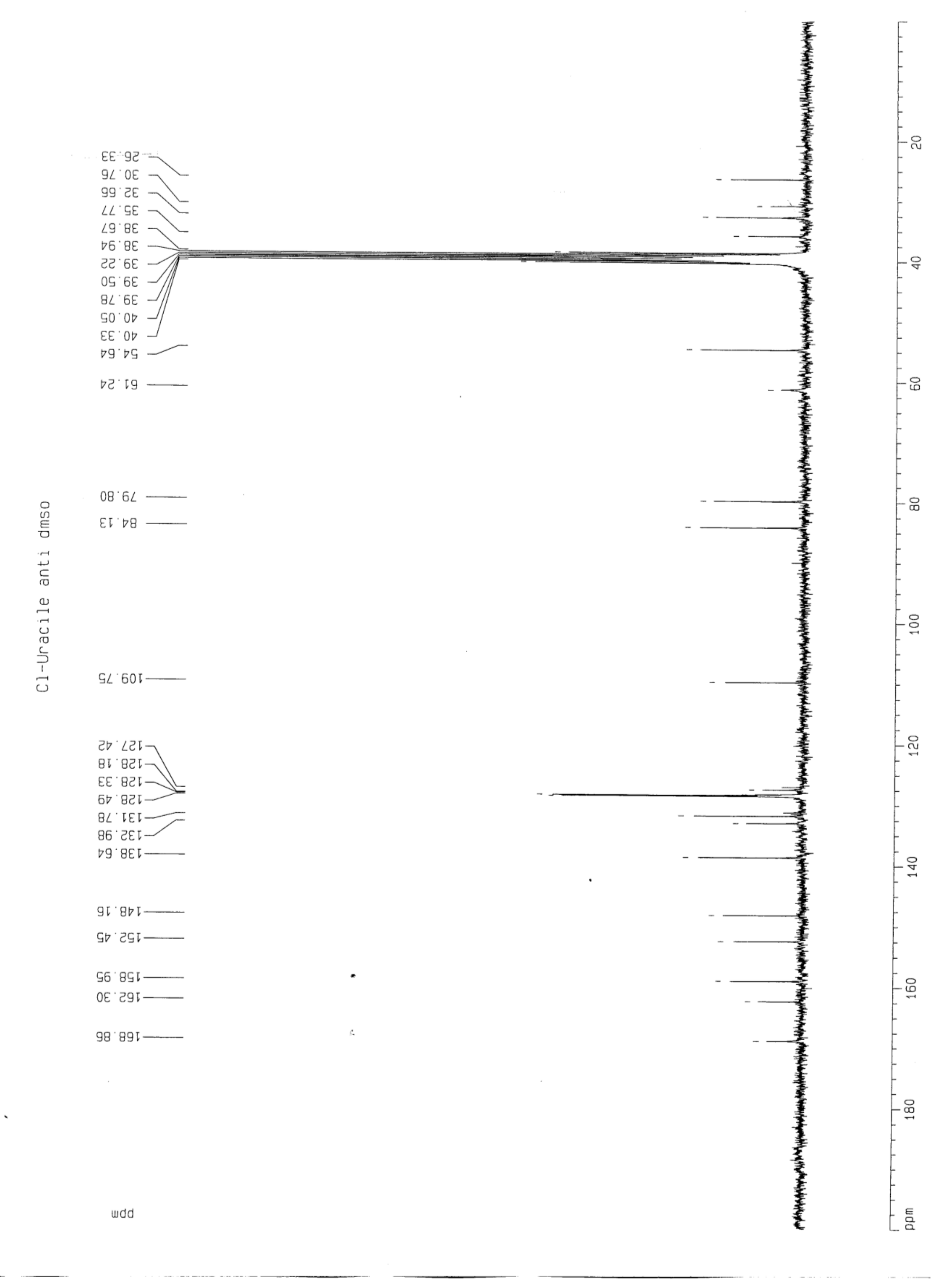
Cl-Uracile anti dmso
26.33
30.76
32.66
35.77
38.67
38.94
39.22
39.50
39.78
40.05
40.33
54.64
61.24
79.80
84.13
109.75
127.42
128.18
128.33
128.49
131.78
132.98
138.64
148.16
152.45
158.95
162.30
168.86
ppm
20
40
60
80
100
120
140
160
180
ppm

6aC.

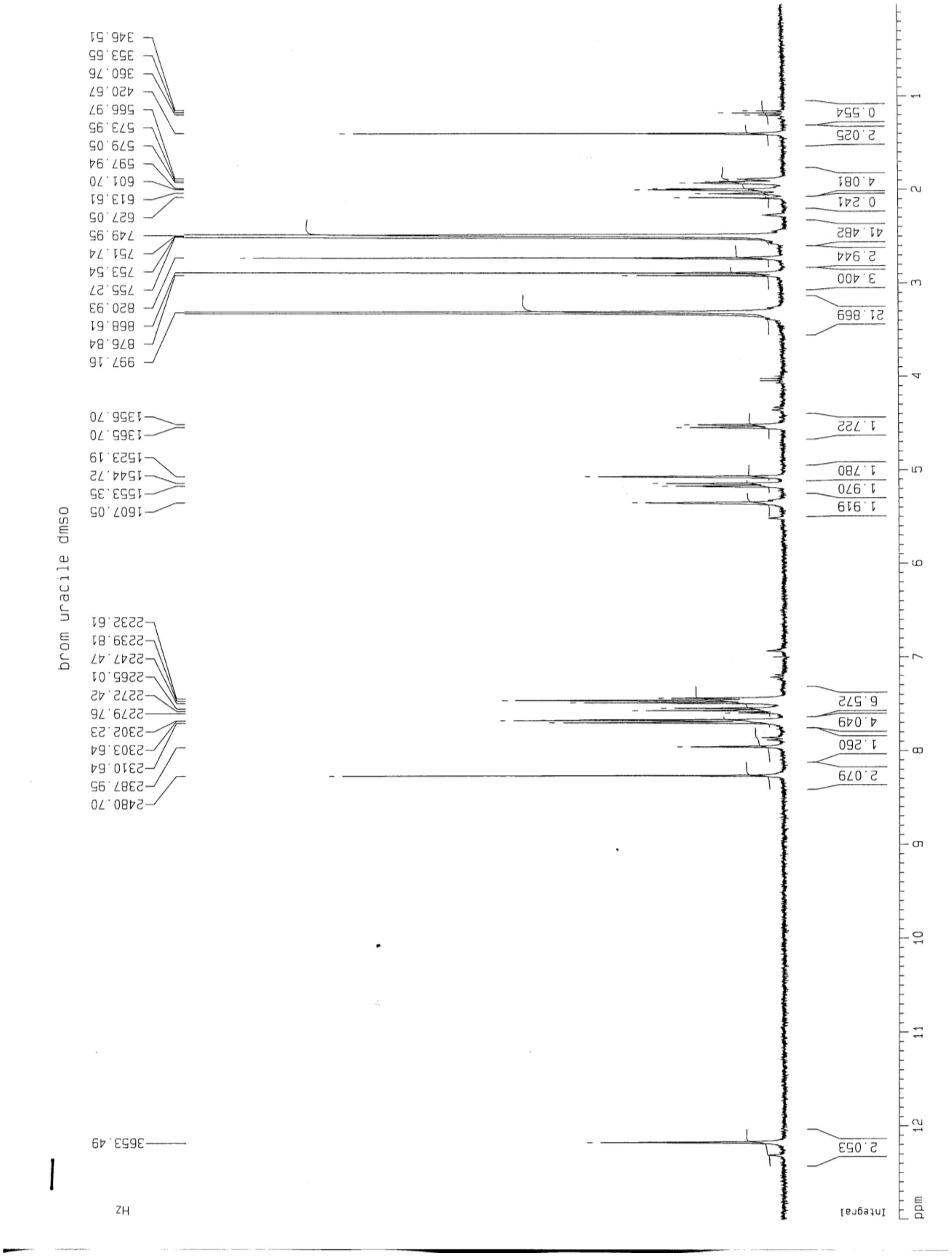

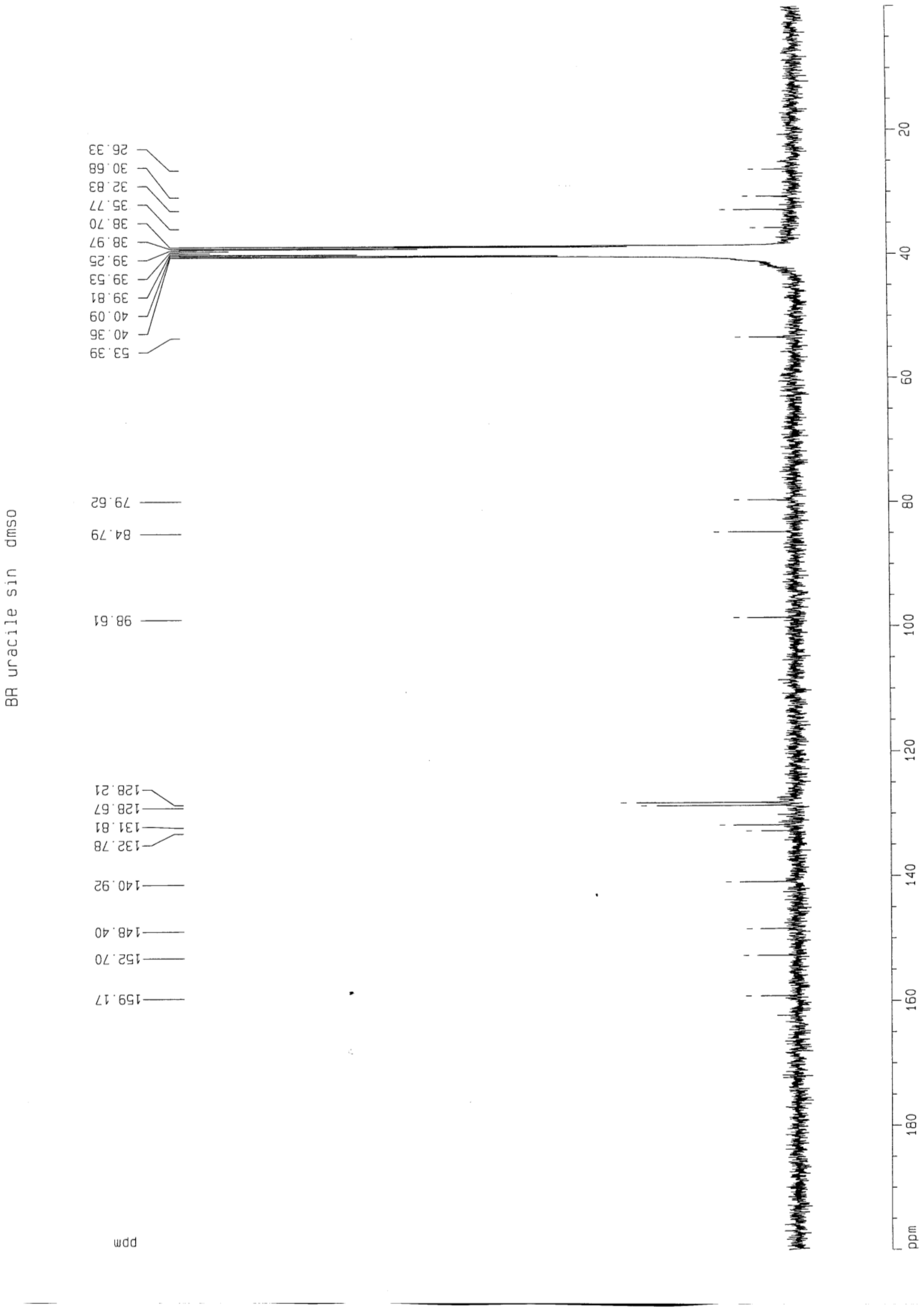
BR uracile sin dmso
26.33
30.68
32.83
35.77
38.70
38.97
39.25
39.53
39.81
40.09
40.36
53.39
79.62
84.79
98.61
128.21
128.67
131.81
132.78
140.92
148.40
152.70
159.17
ppm
20
40
60
80
100
120
140
160
180
ppm

6bC.

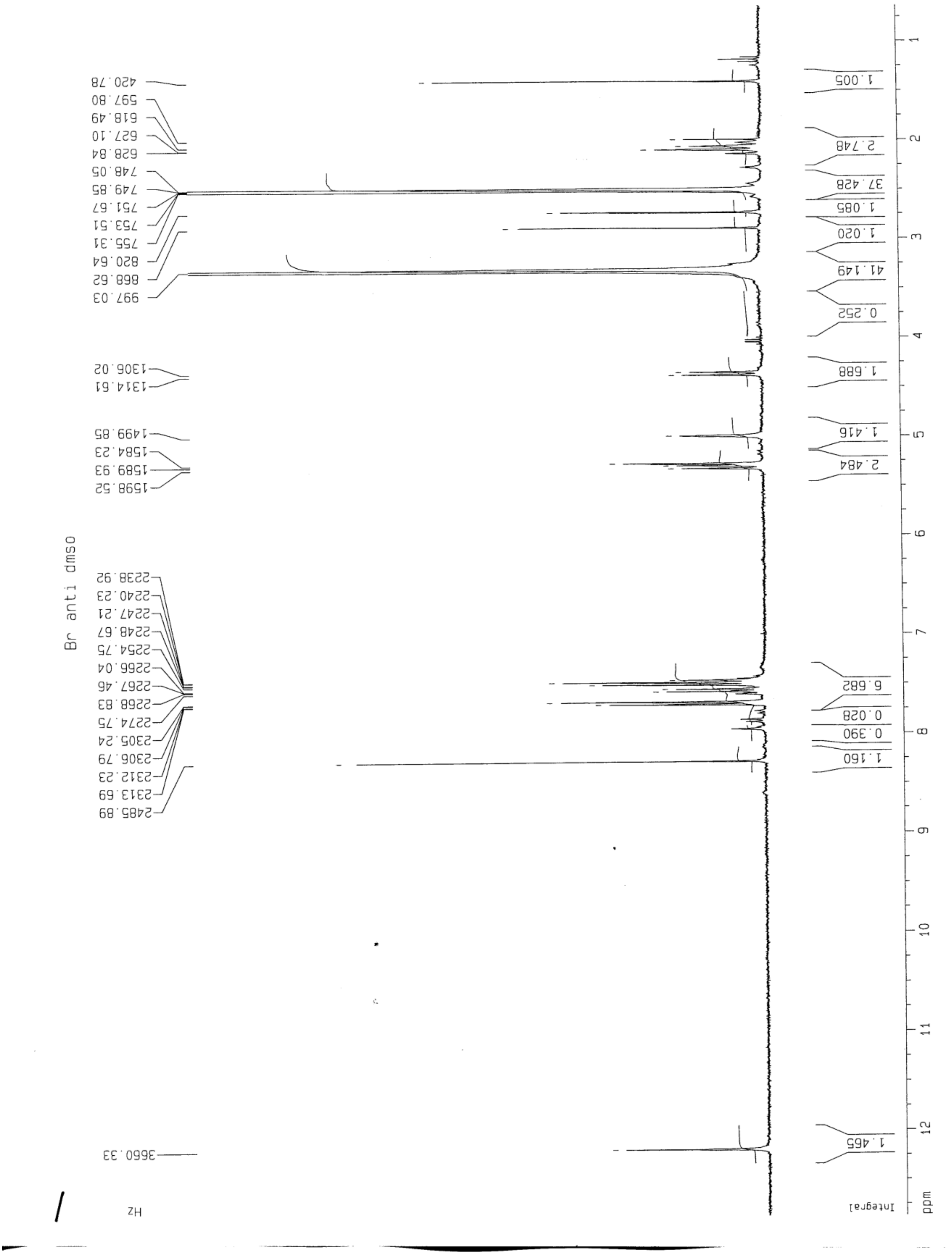
Br anti dmso
420.78
597.80
618.49
627.10
628.84
748.05
749.85
751.67
753.51
755.31
820.64
868.62
997.03
1306.02
1314.61
1499.85
1584.23
1589.93
1598.52
2238.92
2240.23
2247.21
2248.67
2254.75
2266.04
2267.46
2268.83
2274.75
2305.24
2306.79
2312.23
2313.69
2485.89
3660.33
Hz
1.005
2.748
37.428
1.085
1.020
41.149
0.252
1.688
1.416
2.484
6.682
0.028
0.390
1.160
1.465
Integral
1
2
3
4
5
6
7
8
9
10
11
12
ppm

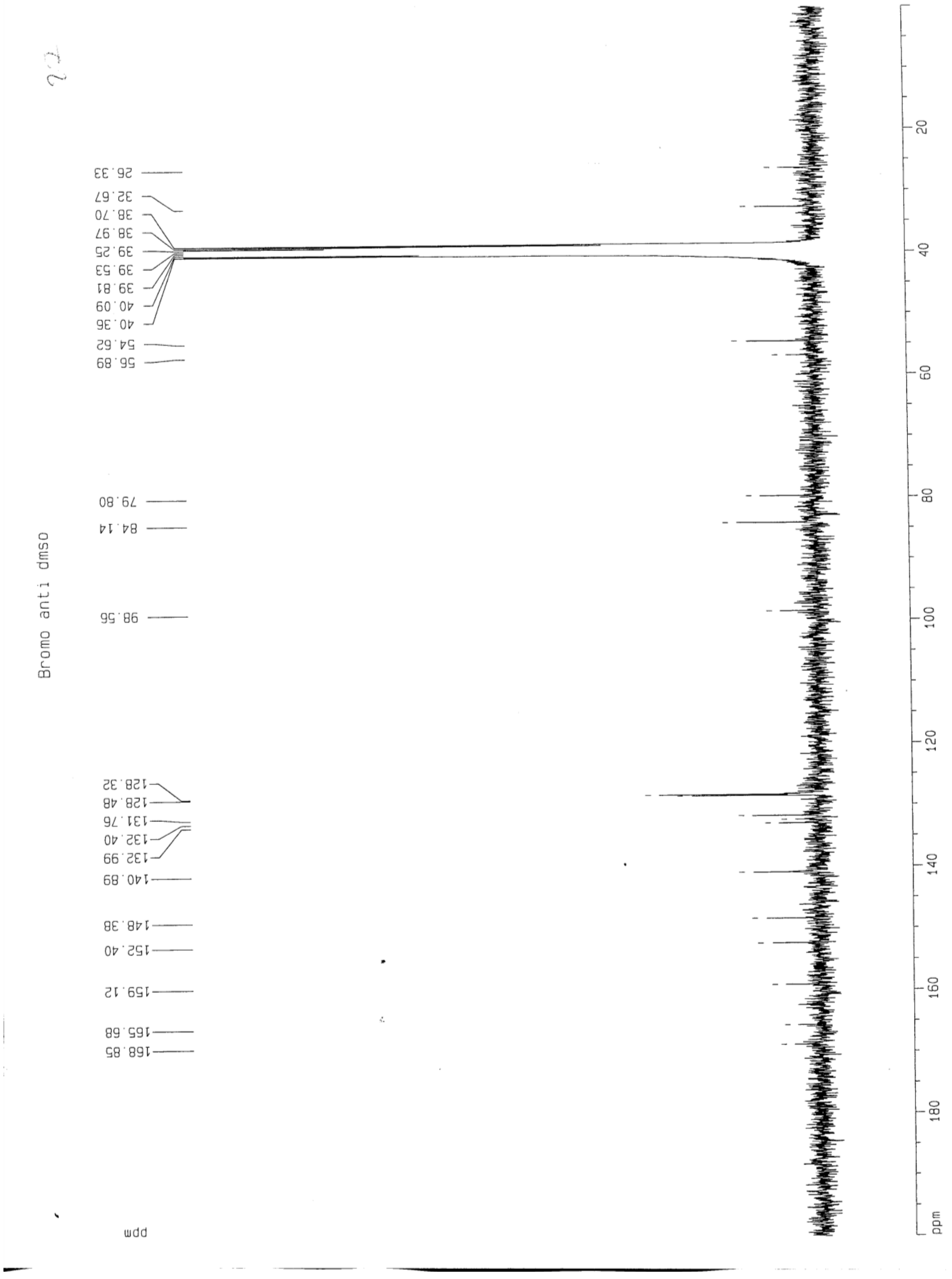
Bromo anti dmso
26.33
32.67
38.70
38.97
39.25
39.53
39.81
40.09
40.36
54.62
56.89
79.80
84.14
98.56
128.32
128.48
131.76
132.40
132.99
140.89
148.38
152.40
159.12
165.68
168.85
ppm
20
40
60
80
100
120
140
160
180
ppm

6aD.

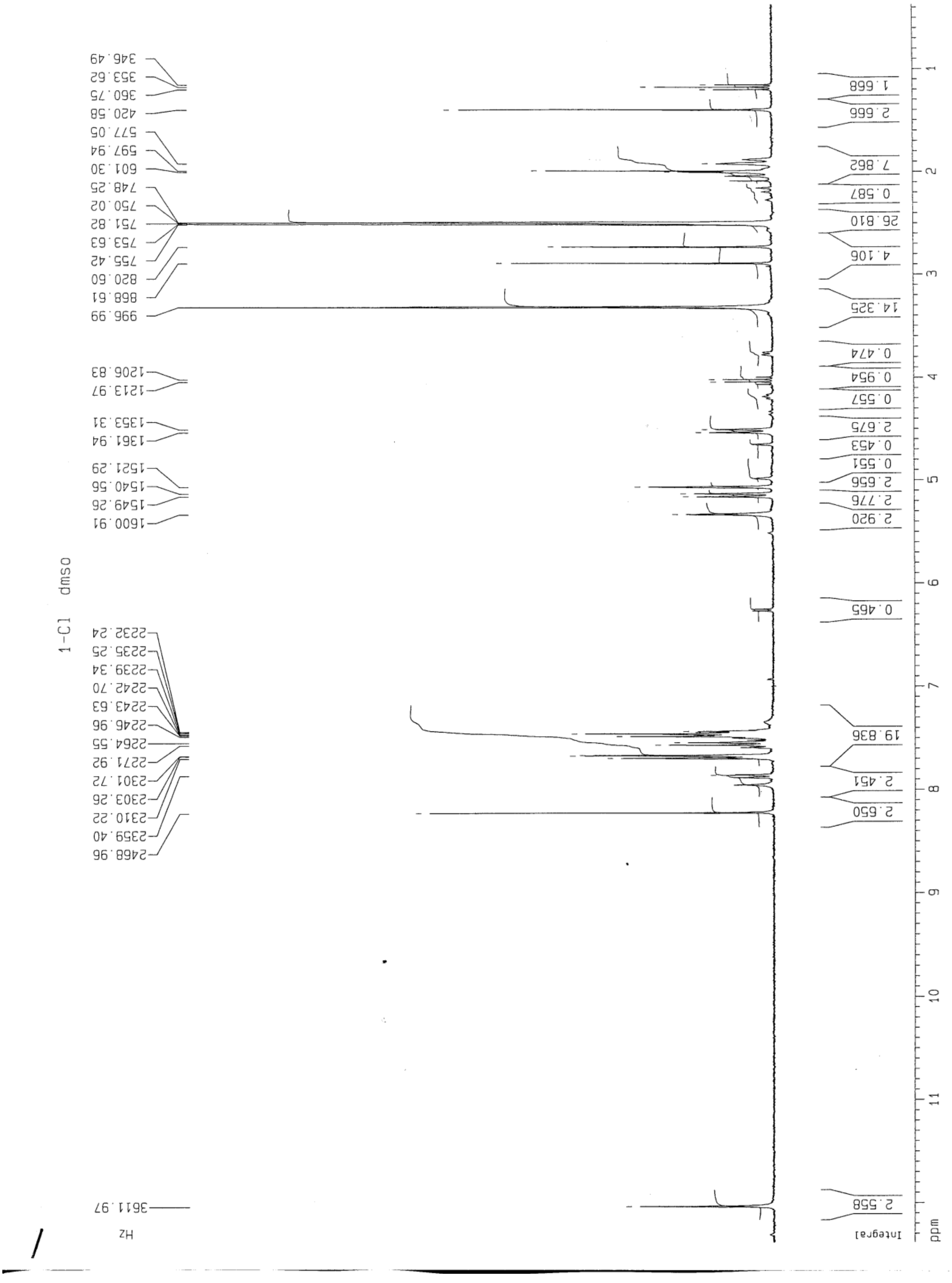

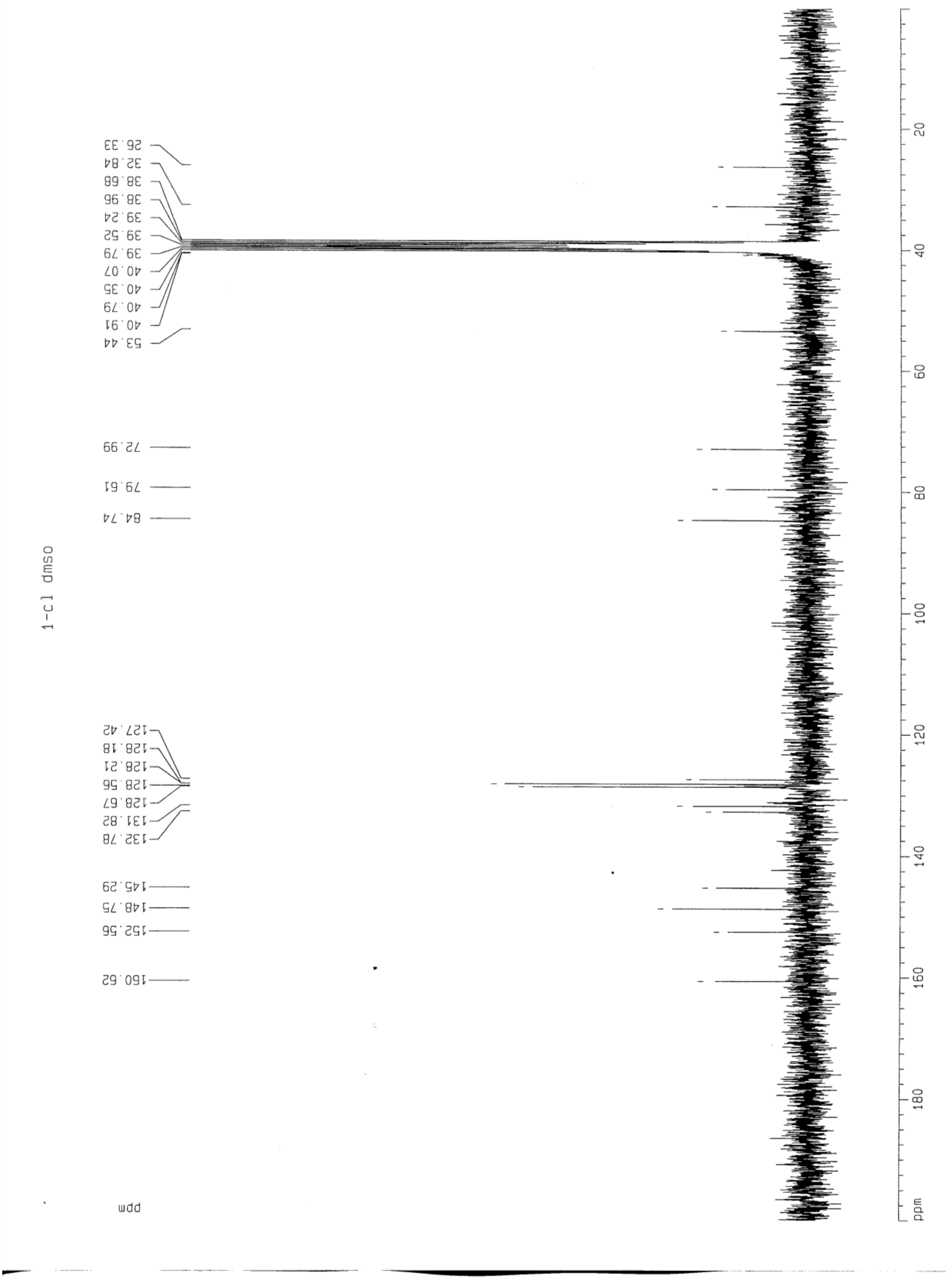
1-cl dmso
26.33
32.84
38.68
38.96
39.24
39.52
39.79
40.07
40.35
40.79
40.91
53.44
72.99
79.61
84.74
127.42
128.18
128.21
128.56
128.67
131.82
132.78
145.29
148.75
152.56
160.62
ppm
20
40
60
80
100
120
140
160
180
ppm

6bD.

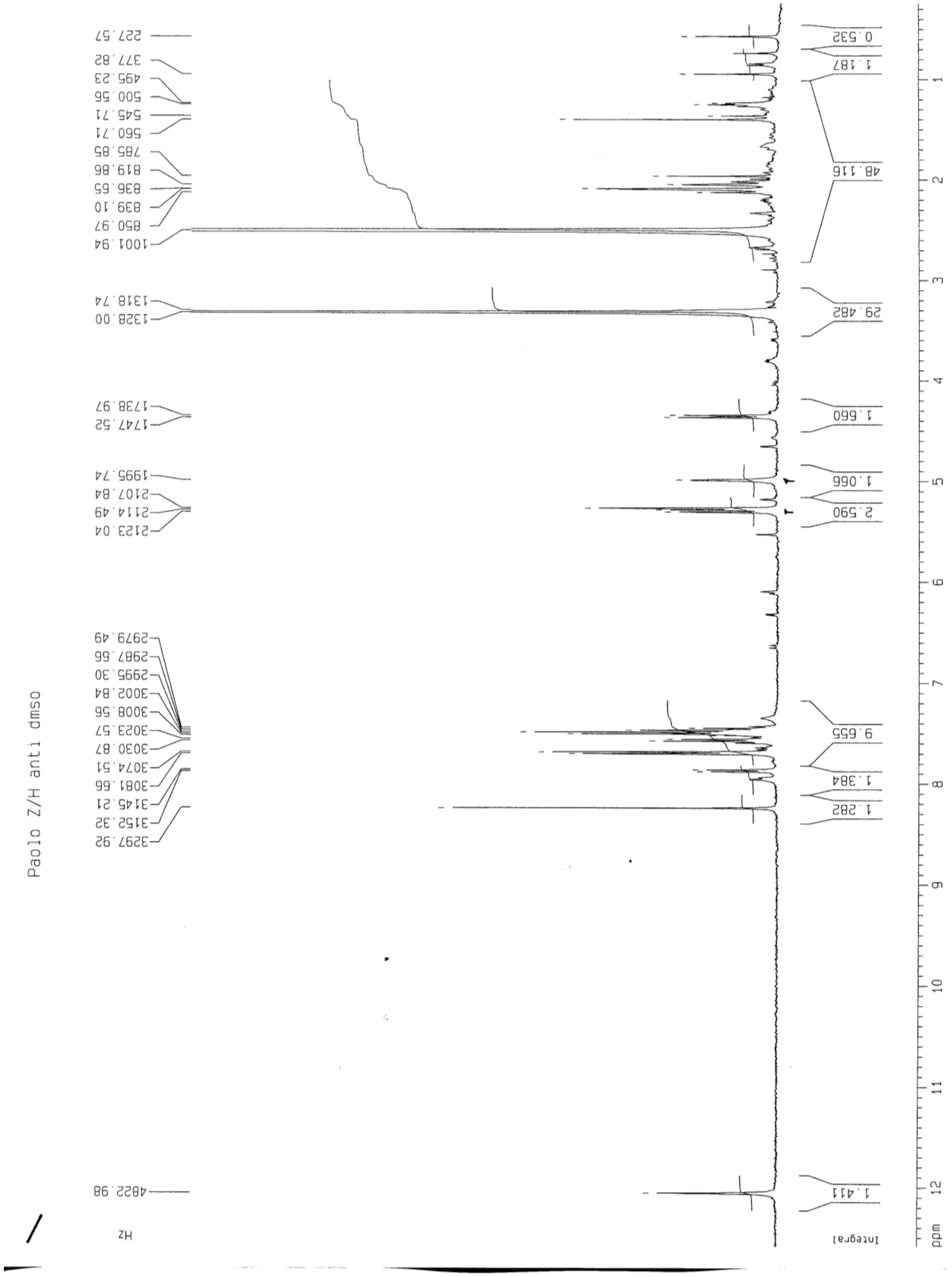
Paolo Z/H anti dmso
227.57
377.82
495.23
500.56
545.71
560.71
785.85
819.86
836.65
839.10
850.97
1001.94
1318.74
1328.00
1738.97
1747.52
1995.74
2107.84
2114.49
2123.04
2979.49
2987.66
2995.30
3002.84
3008.56
3023.57
3030.87
3074.51
3081.66
3145.21
3152.32
3297.92
4822.98
Hz
0.532
1.187
48.116
29.482
1.660
1.066
2.590
9.655
1.384
1.282
1.411
Integral
ppm

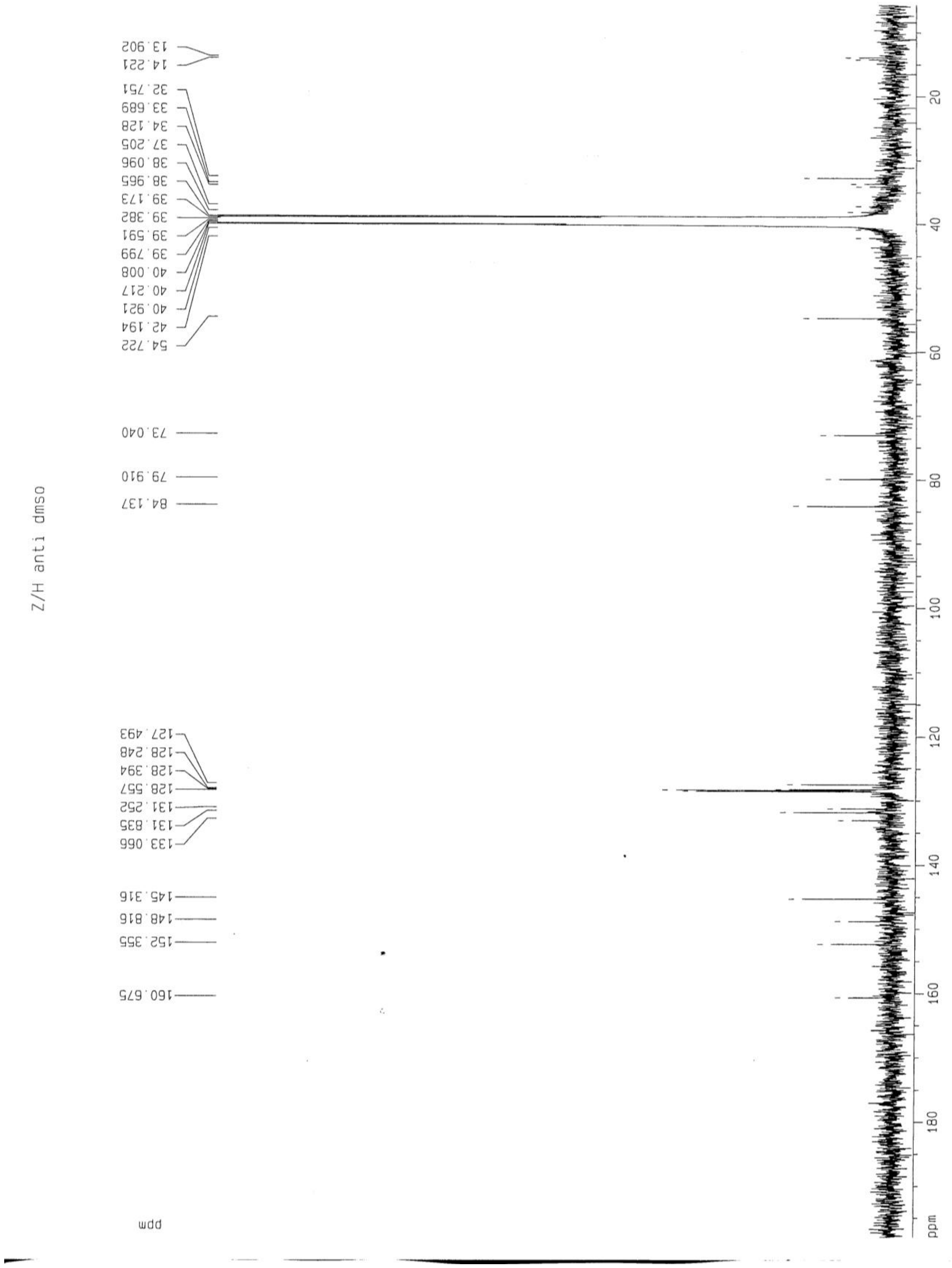
Z/H anti dmso
13.902
14.221
32.751
33.689
34.128
37.205
38.096
38.965
39.173
39.382
39.591
39.799
40.008
40.217
40.921
42.194
54.722
73.040
79.910
84.137
127.493
128.248
128.394
128.557
131.252
131.835
133.066
145.316
148.816
152.355
160.675
ppm

7a.

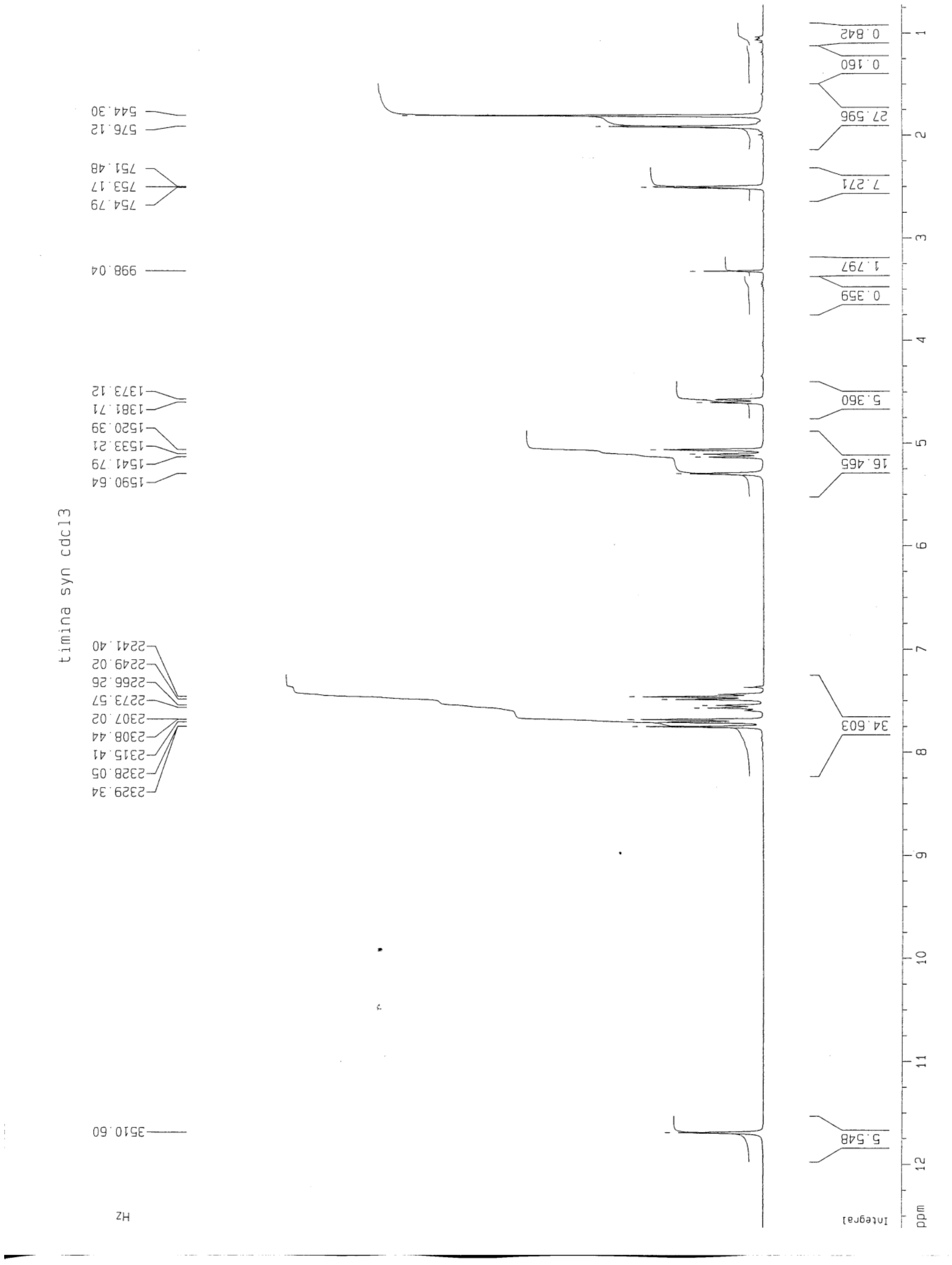
timina syn cdcl3
544.30
576.12
751.48
753.17
754.79
998.04
1373.12
1381.71
1520.39
1533.21
1541.79
1590.64
2241.40
2249.02
2266.26
2273.57
2307.02
2308.44
2315.41
2328.05
2329.34
3510.60
Hz
0.842
0.160
27.596
7.271
1.797
0.359
5.360
16.465
34.603
5.548
Integral
ppm

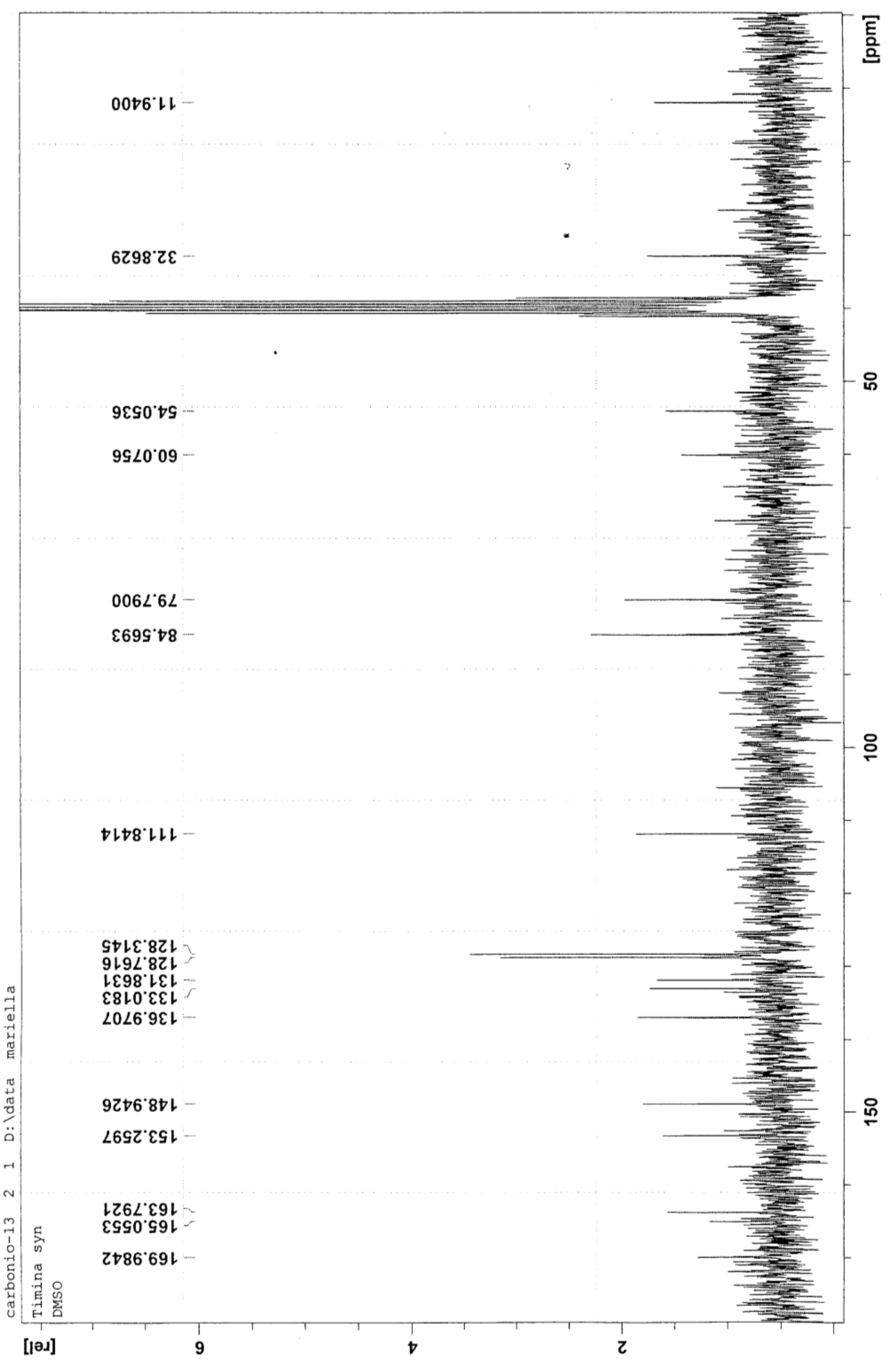
carbonio-13 2 1 D:\data mariella
Timina syn
DMSO
169.9842
165.0553
163.7921
153.2597
148.9426
136.9707
133.0183
131.8631
128.7616
128.3145
111.8414
84.5693
79.7900
60.0756
54.0536
32.8629
11.9400
[ppm]
[rel]

7b.

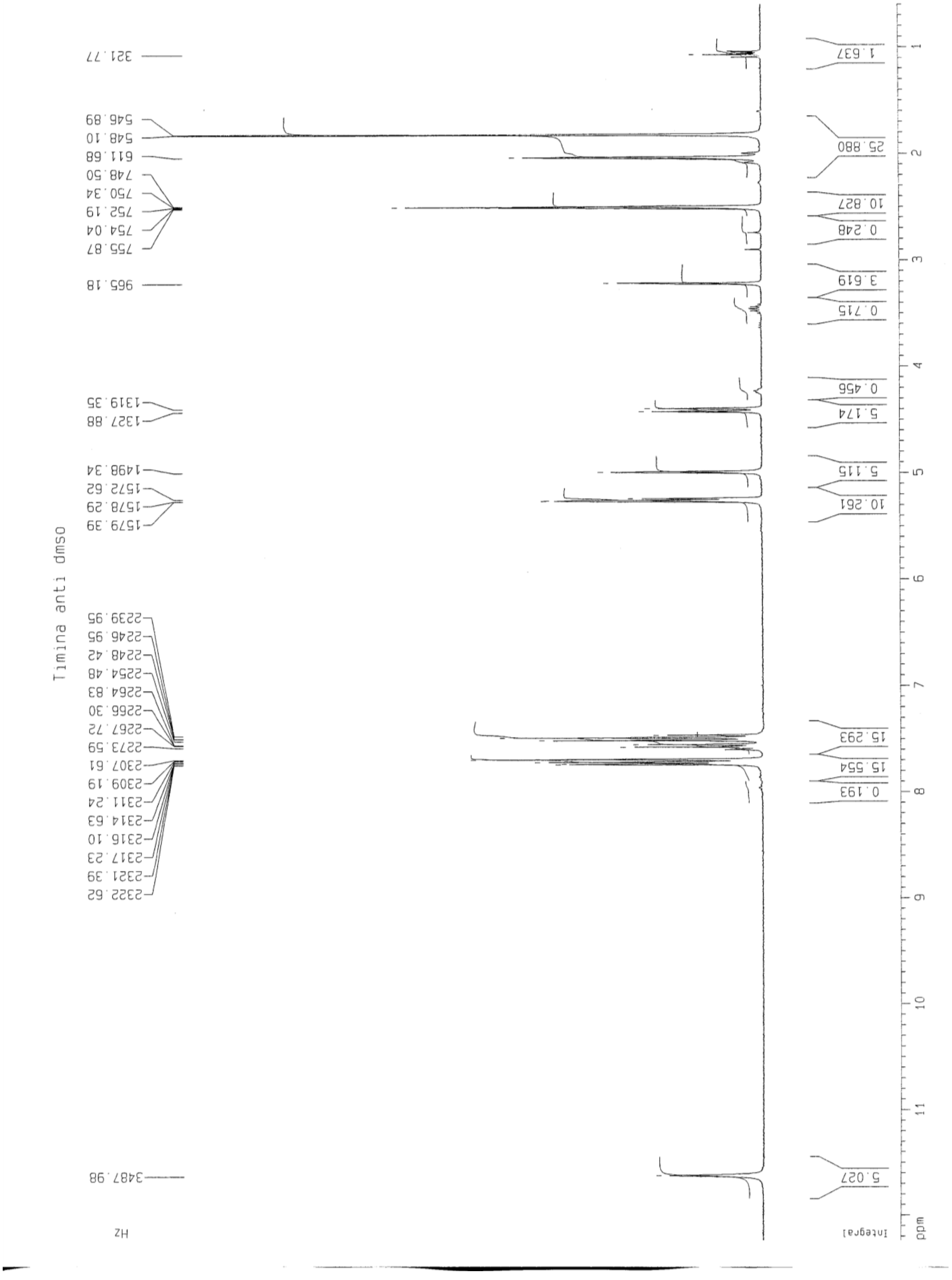
Timina anti dmso
321.77
546.89
548.10
611.68
748.50
750.34
752.19
754.04
755.87
965.18
1319.35
1327.88
1498.34
1572.62
1578.29
1579.39
2239.95
2246.95
2248.42
2254.48
2264.83
2266.30
2267.72
2273.59
2307.61
2309.19
2311.24
2314.63
2316.10
2317.23
2321.39
2322.62
3487.98
Hz
1.637
25.880
10.827
0.248
3.619
0.715
0.456
5.174
5.115
10.261
15.293
15.554
0.193
5.027
Integral
1
2
3
4
5
6
7
8
9
10
11
ppm

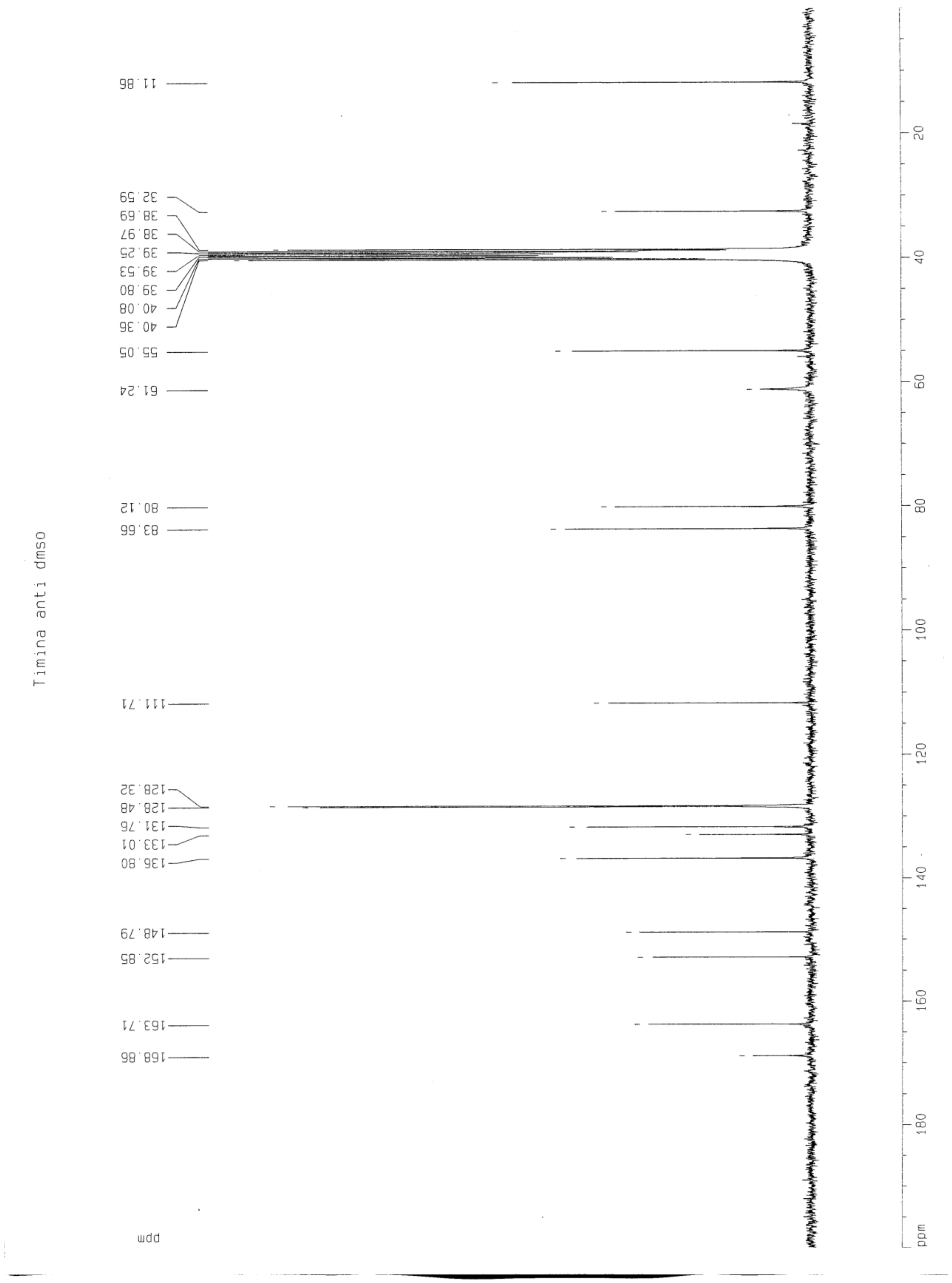
Timina anti dmso
11.86
32.59
38.69
38.97
39.25
39.53
39.80
40.08
40.36
55.05
61.24
80.12
83.66
111.71
128.32
128.48
131.76
133.01
136.80
148.79
152.85
163.71
168.86
ppm
20
40
60
80
100
120
140
160
180
ppm
